# Chronic administration of prebiotics and probiotics prevent pathophysiological hallmarks of Alzheimer’s disease in the cortex of APP/PS1 mice

**DOI:** 10.3389/fphar.2025.1596469

**Published:** 2025-05-15

**Authors:** Giorgia Sarti, Chiara Traini, Giada Magni, Selene Attorre, Giorgio Tognozzi, Edoardo Calussi, Maria Grazia Giovannini, Maria Giuliana Vannucchi, Daniele Lana

**Affiliations:** ^1^ Department of Experimental and Clinical Medicine, Research Unit of Histology and Embryology, University of Florence, Florence, Italy; ^2^ Cnr-Institute of Applied Physics “Nello Carrara”, Sesto Fiorentino, Italy; ^3^ Section of Pathological Anatomy, Department of Health Sciences, University of Florence, Florence, Italy; ^4^ Section of Pathological Anatomy, Careggi University Hospital, Florence, Italy; ^5^ Department of Health Sciences, Section of Clinical Pharmacology and Oncology, University of Florence, Florence, Italy

**Keywords:** Alzheimer’s disease, astrocytes, neurodegeneration, neuritic plaques, beta-amyloid, microbiota, ball-and-chain microglia

## Abstract

**Introduction:**

Dysbiosis is a characteristic of patients with Alzheimer's disease (AD). The disbalance between Gram-negative and Gram-positive bacteria causes increased production of beta-amyloid (Aβ) in the gut, which can contribute to brain accumulation of Aβ. Recovering microbiota composition with symbiotic administration of prebiotics and probiotics may be a strategy to prevent or reduce AD symptomathology. The aim of this research was to study whether chronic administration of pre- and probiotics modifies the histopathological signs of neurodegeneration in the cortex of APP/PS1 mice, a transgenic mouse model of AD. We focused on neuritic plaques deposition, neuronal degeneration and glia activation.

**Methods:**

Transgenic (TG) mice and Wild type (WT) littermates were fed daily with a diet supplemented with prebiotics (a multi-extract of fibers and plant complexes, containing inulin/fruit-oligosaccharides) and probiotics (a 50%–50% mixture of Lactobacillus rhamnosus and Lactobacillus paracasei). The treatment started at 2 months of age and lasted for 6 months. Controls were WT and TG mice fed with a standard diet. All groups were evaluated qualitatively and quantitatively by immunofluorescence, confocal microscopy and digital imaging. Cortical sections were immunostained for neuritic plaques, neurons, astrocytes, microglia, and inflammatory proteins. Qualitative and quantitative analyses were carried out by immunofluorescence, confocal microscopy and digital imaging with ImageJ software.

**Results:**

Quantitative analyses in TG mice demonstrated intense Aβ load and accumulation of neurofilament heavy polypeptide (NHP) in neuritic plaques, neuronal degeneration, shrinkage of the cortex, increase of GFAP expression, and microglia and astrocytes activation. All these effects were mainly evident in cortical Layer 5. The symbiotic treatment with pre- and probiotics decreased Aβ deposition and neuritic plaques in the frontoparietal cortex. In addition, the treatment decreased the degeneration of neurons, the cortical shrinkage, increased GFAP expression, and modified microglia phenomic, decreasing significantly microglia activation. The abovementioned effects of the treatment were mostly evident in cortical Layer 5.

**Discussion:**

These data confirm that prolonged dietary regimen enriched with pre- and probiotics counteracts many of the histopathological hallmarks of AD, and poses the bases for a simple, affordable treatment that may help prevent AD.

## Introduction

Alzheimer’s Disease (AD), a neurodegenerative disorder characterized by the slow, irreversible decline of memory, affects 40 million people in Western countries, with a prevalence expected to increase in the next few decades, with the aging of the population. Since the complete understanding of the pathophysiology of AD is so far an unmet need, effective treatments that prevent, slow down or cure the disease are still lacking. Precipitation and accumulation of abnormal proteins in the brain are the pathophysiological mechanisms of many neurodegenerative diseases such as AD (Jucker and Walker, 2018). Indeed, the accumulation of misfolded beta-amyloid (Aβ) peptides that precipitate and form plaques in the central nervous system (CNS) parenchyma is one of the histopathological hallmarks of AD (Glenner and Wong, 1984; Koyama et al., 2012).

The gut and its microbiota (MB-gut) are major sites of Aβ production outside the brain (Galloway et al., 2019; Pallebage-Gamarallage et al., 2012), and quantitative and qualitative modifications of gut microbiota cause aberrant accumulation of amyloid precursor protein (APP) in the gut (Brandscheid et al., 2017; Kowalski and Mulak, 2019). It has been demonstrated that patients affected by AD, even at the early stages of the disease, have dysbiosis (Vogt et al., 2017; Kesika et al., 2021; Sarkar and Biswas, 2021), mainly characterized by increased Gram-negative and decreased Gram-positive bacteria strains in the gut. Intestinal Aβ aberrant deposition is also detected in autopsies of AD patients (Liu et al., 2020).

Transgenic animal models of AD such as the double transgenic mice expressing a chimeric mouse/human amyloid precursor protein (Mo/HuAPP695swe) and a mutant human presenilin 1 (PS1-dE9) (APP/PS1) mice, have dysbiosis, with increased relative abundance of *B. subtilis* (*Bacillus subtilis*) and *E. coli (Escherichia coli)* (Harach et al., 2017), large producers of Aβ. In addition, *E. coli* produces an endotoxin that promotes the formation of Aβ fibrils. Gram-negative strains are likely implicated in the pathogenesis of neurodegeneration (Brown, 2019; Qian et al., 2021). On the other hand, a decrease of Gram-positive strains, mostly Lactobacilli that produce trophic agents for the gut and the brain such as short-chain fatty acids (SCFAs) and tryptophan, was demonstrated in AD animal models (Pistollato et al., 2016; Sharon et al., 2016; Cattaneo et al., 2017; Friedland and Chapman, 2017; Vogt et al., 2017; Mancuso and Santangelo, 2018; Galloway et al., 2019; Giovannini et al., 2021; Willyard, 2021).

Dysbiosis causes dysfunction of the intestinal barrier (Iadecola et al., 1999), which may activate intestinal innate immunity before the onset of CNS neuroinflammation (Semar et al., 2013). Dysbiosis may also cause dysfunction of the blood brain barrier (BBB) (Mayer and Tillisch, 2011; Sharon et al., 2016; Brown, 2019). When Aβ reaches the brain through the circulation, its overload or defective clearance may cause its accumulation, promoting fibril organization and plaque deposition (Brown, 2019).

The eubiotic gut microbiota produces molecules necessary for the correct physiological functions of the brain (Sharon et al., 2016; Strandwitz, 2018). However, how dysbiosis contributes to neurodegeneration remains to be completely understood, but accumulating evidence demonstrates that in transgenic APP/PS1 mice with dysbiosis, the levels of Aβ in the CNS increase and spatial learning and memory is impaired (Shen et al., 2017; Traini et al., 2024). Indeed, neuronal loss and memory impairment in AD are mainly associated with Aβ plaques in brain parenchyma and with intracellular deposits of hyperphosphorylated tau.

Maintaining or restoring the eubiosis of the gut microbiota seems to be fundamental to keeping under control Aβ production and avoiding its deposition in the brain. Studies on the effects of prebiotic or probiotic, administered separately in the diet, on Aβ deposition, neurodegeneration and cognitive functions and brain damage have obtained encouraging results (Pantano et al., 2017; Bonfili et al., 2018; 2021; Ou et al., 2020; Sun et al., 2020; Zhu et al., 2021; Wu et al., 2023). Nevertheless, very few studies have been carried out on the effects of the simultaneous, symbiotic administration of prebiotics and probiotics on these parameters in animal models of AD.

Astrocytes establish millions of contacts with synapses and vascular capillaries to modulate vital functions, maintain homeostasis, and provide trophic support to neurons (Schiweck et al., 2018). Alterations of the BBB, caused by dysbiosis, allow the passage of peptides such as Aβ, of proinflammatory factors, and of immune cells from the periphery to the brain, changing the composition of the brain microenvironment and brain cell homeostasis. The gut microbiota can modulate and suppress the inflammatory state of astrocytes, with important consequences for neuroinflammation (Rothhammer et al., 2018). An in-depth characterization of the mechanisms that control astrocytes and the role of prebiotics and probiotics on their phenotypic modification is still lacking.

The presence of microglia around plaques and their interaction with Aβ has been extensively documented in both AD patients and in transgenic mouse models of AD (Stalder et al., 1999; Serrano-Pozo et al., 2014), but their exact role, whether damaging or protective, is not yet understood. Indeed, activation of microglia seems to play a dual role in the pathogenesis of AD: firstly, microglia activate to increase phagocytosis of Aβ and reduce its accumulation, but activated microglia at later stages, triggering proinflammatory cascades, likely contribute to neurotoxicity and synapse loss (Sarlus and Heneka, 2017).

Nevertheless, astrocytes and microglia in different regions of the brain do not behave equally in response to the same damaging insult, and their responses differ markedly. It has been demonstrated that different microglia, astrocytes and oligodendrocytes subpopulations from the same cortical portion of aged individuals have different AD-related traits (Green et al., 2024). Averting the polarization of astrocytes, microglia and oligodendrocytes into specific phenomics may give new perspectives for therapeutic interventions on AD (Green et al., 2024). However, studies aimed at clarifying the relationship between gut dysbiosis, gut and brain Aβ accumulation, glia phenomic modification and neuronal survival in AD animal models are few.

In recent published papers from our laboratory, we developed a protocol to administer pre- and probiotics simultaneously through the diet to APP/PS1 mice (Lana et al., 2024; Traini et al., 2024), with the idea that prebiotics sustain the survival of probiotic Lactobacilli co-present in the preparation, and are substrates of the Firmicutes, a strain that decreases with age (Cattaneo et al., 2017; Galloway et al., 2019; Sun et al., 2020). We demonstrated in APP/PS1 mice (Traini et al., 2024) that treatment with pre- and probiotics modifies the bacteria strain imbalance in the gut microbiota, restores mucous secretion, and reduces Aβ blood levels and ameliorates the memory deficits. In the hippocampus, the treatment decreases Aβ plaque deposition, protects neurons, causes modification of astrocytes and microglia phenomic, mainly in CA3, and to a lesser extent in CA1 (Lana et al., 2024).

Based on these previously published findings (Lana et al., 2024; Traini et al., 2024) we used brain sections from the same animal groups to further explore the effects of the diet enriched in prebiotics and probiotics on the hallmarks of AD and on the alterations of neurons and glia in the cerebral cortex of APP/PS1 mice.

The results of this research will help to understand the beneficial effects of pre- and probiotics administration starting early in life on the hallmarks of AD pathogenesis in the cortex. As diet is one of the most effective approaches to modify the microbiota, food-based therapies, influencing its composition, can modify the function of the CNS with very few, if any, side effects. Specific individualized nutritional interventions could be an effective strategy to modify Aβ production and aggregation.

## Materials and methods

### Animals

Male and female APP/PS1 transgenic mice aged 8 months were used as the disease group and littermate C57BL/6 wild-type mice aged 8 months were used as the control group. The mice were bred and treated by the team of Prof. Vannucchi’s lab.

The APP/PS1 mouse model express human APP with the Swedish mutation (Mo/HuAPP695swe) and L166P-mutated human presenilin1 (PS1) under the control of a neuron-specific Thy1 promoter (Radde et al., 2006; Rupp et al., 2011).

All mice were housed under a 12-h light/12-h dark cycle with food and water available *ad libitum*. The room temperature was kept at around 22°C.The experimental protocols were approved by the Italian Ministry of Health (code: 53/2022) and were carried out in accordance with the European Communities Council Directive of 2010/63/EU. The authors further attest that all efforts were made to minimize the number of animals used and their suffering, as reported in the Guidelines McGill Module 1.

### Animal treatments

The probiotic chosen is a 50% mixture of each type of the following bacteria: *Lactobacillus rhamnosus* IMC 501 and *Lactobacillus paracasei* IMC 502, supplied by Synbiotec s.r.l (Camerino, MC). The probiotic was dispersed in gelled water through the addition of an instant powdered cornstarch thickener (4.5 g/100 mL), a tasteless and non-toxic substance. The dose of probiotic administered was 1.8*10^8^ CFU/day/25 g mouse, normalized to the body surface area starting from the recommended dose in humans (15 × 10^9^ CFU*2/day) as described in the literature (Gui et al., 2015). The probiotic was dispersed directly in water and supplied fresh daily. The probiotic concentration was adjusted to the weight gain of the animals and the volume of water intake daily.

The treatments consisted of an enrichment of the daily diet with prebiotics and probiotics, lasted for 6 months, and were administered from the completion of the second month until the eighth month of age. The mode of diet administration did not result in stress for the animal. The treatment, described in more detail in the previous article by Traini and colleagues (Traini et al., 2024), was as follows.

#### Prebiotic formulation and administration

The prebiotic was a multi-extract of fibers and plant complexes, mainly composed of inulin/FOS (fruit oligosaccharides), purchased from the company Aboca (San Sepolcro, AR, Italy). The chosen dosage (50 mg inulin/FOS/g diet) corresponded to a 5% increase in inulin/FOS in comparison to the standard diet. This percentage is consistent with the literature (Mao et al., 2015) and with the dosage commonly suggested as a dietary supplement for humans. The pellets containing the mix of the standard diet plus prebiotics retained the taste of the standard diet. The pellets were tailor-made, prepared by Mucedula s.r.l. (Milan, Italy) and purchased from them. The food intake was checked daily for the entire period of administration, weighing the leftovers and replenishing the food pellets.

#### Probiotic formulation and administration

The probiotic chosen was a 50% mixture of each type of the following bacteria: *Lactobacillus rhamnosus* IMC 501 and *Lactobacillus paracasei* IMC 502, supplied by Synbiotec s.r.l (Camerino, MC, Italy). The probiotic formulation was supplied without excipients or prebiotics, with a bacterial load corresponding to 100 billion cells/g. The chosen dosage was 1.8 × 10^8^ CFU/day/25 g per mouse. It was calculated following the normalization method with respect to the body surface area and considering the recommended human dose (15 × 10^9^ CFU*2/day). The dosage was consistent with the literature (Gui et al., 2015). As demonstrated by Verdenelli and colleagues (Verdenelli et al., 2009), the chosen bacteria survive at gastric pH, tolerate bile acid, do not generate any side effects, have high adhesion and colonization ability, and are recovered in colonic fecal samples. The viability of the bacteria at room temperature is guaranteed up to 12 h. The probiotics were dispersed in drinking water gelled by adding an instant thickener powder made from cornstarch (2.5 g/100 ml) that is tasteless, devoid of toxicity, and commonly used for swallowing deficiencies in humans. This mode of administration is stress-free for the animal and guarantees a uniform redistribution of the probiotics in the water. The volume of gelled water containing the probiotics was prepared and supplied fresh every day for the entire period of treatment. The gelled water was placed into a Nombrero (Animal Specialties and Provisions, LLC, Quakertown, PA, United States), a container specifically designed to feed rodents with wet food that has proven to be particularly suitable for containing gelled water. The presence of a hook made it possible to hang the Nombrero in the upper part of the cage, so that it would not tip over and the gelled water would not get in contact with the litter in the cage, limiting its contamination. However, the basal part, containing the gelled water, was shallow enough to be easily accessible by the mice. The amount of water taken was assessed every day by weighing the residual water. The probiotic concentration was adjusted to the mice weight gain and the amount of water consumed. As rodents drink mainly during the night, the water containing the probiotics was administered every evening, and the following morning the residual water was weighed. To guarantee similar environmental conditions, all the animals received gelled drinking water, with or without probiotics.

### Animal groups

The animals were divided randomly into 4 experimental groups:

Wild type mice, C57BL/6, fed with a standard diet, 3 males and 6 females (WT);
Wild type mice, C57BL/6, fed with the standard diet enriched in prebiotics and probiotics, 4 males and 4 females (WT-T);
Transgenic mice, APP/PS1, fed with a standard diet, 4 males and 6 females (TG);
Transgenic mice, APP/PS1, fed with the standard diet enriched in prebiotics and probiotics, 4 males and 6 females (TG-T).

### Sample preparation

At the end of the treatment, the mice were anesthetized with ketamine–dexmedetomidine, s.c. (80–120 mg/kg + 0.5–1.0 mg/kg, respectively). One hemibrain was rapidly dissected, immediately placed in ice-cold 4% paraformaldehyde in 0.1 M phosphate-buffered saline (PBS, pH 7.4) and fixed overnight (ON) at 4°C. The fixed hemibrain was then placed in cold 30% sucrose/PBS and after 48 h it was immersed in a solution of isopentane at −40°C until the hemibrain was completely frozen. Coronal sections of 40 μm thickness were cut with a cryostat and stored at −20°C in anti-freeze solution (40% PBS,30% ethylene-glycol, 30%, glycerol, v/v) until immunohistochemistry.

### Immunohistochemistry

Immunohistochemistry was performed with the free-floating method (Giovannini et al., 2002; Lana et al., 2014) on mice hemibrain coronal sections cut at −1700 µm posterior from Bregma. The antibodies used (see Table 1) and the protocols of the different immunostainings are reported below.

**TABLE 1:** Antibodies used for immunohistochemistry. All antibodies were diluted in BB solution.

Antibodies used for immunohistochemistry							
Target	Antigen	Supplier	Catalog #	Antibody	Host	Usage	Diluition
Neurons	NeuN (Neuronal nuclei)	Millipore (Billerica, MA, United States)	MAB377X	Monoclonal	Ms	Primary Alexa Fluor 488 conjugated	1:100
Beta-amyloid plaques	Aβ (Beta amyloid)	Biolegend (Dedham, MA, United States)	39320–200	Monoclonal	Ms	Primary	1:200
Neurofibrillary tangles and neutitic plaques	Neurofilament Heavy Protein (NHP)	AbCam	ab136407	Polyclonal	Rb	Primary	1:200
Neurons	NeuN (neuronal nuclei)	Millipore	MAB377	Monoclonal	Ms	Primary	1:400
Astrocytes	GFAP (Glial fibrillar acidic protein)	Millipore	MAB3402X	Monoclonal	Ms	Primary Alexa Fluor 488 conjugated	1:500
Total microglia	IBA1 (Ionized calcium-binding adaptor molecule 1)	Wako (Osaka, JP)	016–20001	Policlonal	Rb	Primary	1:300
Total microglia	IBA1 (Ionized calcium-binding adaptor molecule 1)	Wako	011–27991	Policlonal	Gt	Primary	1:200
CD68	CD68 (Cluster of Differentiation 68)	AbCam (Cambridge, UK).	ab125212	Monoclonal	Rb	Primary	1:200
CX3CR1	CX3CR1 (Fraktalkin receptor)	AbCam	ab308613	Monoclonal	Rb	Primary	1:200
Rabbit FC	Rabbit FC	Thermo Fisher (Waltham, MA, United States)	A21206	Polyclonal	Dn	Secondary Alexa Fluor 488	1:400
Mouse FC	Mouse FC	Thermo Fisher	A31570	Polyclonal	Dn	Secondary Alexa Fluor 555	1:400
Rabbit FC	Rabbit FC	Thermo Fisher	A31577	Polyclonal	Gt	Secondary Alexa Fluor 635	1:400
Goat FC	Goat FC	Thermo Fisher	A21082	Polyclonal	Dn	Secondary Alexa Fluor 635	1:400

Before incubation with primary antibodies, all sections were incubated for 60 min with Blocking Buffer (BB) containing 10% Normal Goat Serum (Product Code: S-1000, Vector, Burlingame, CA, United States) in PBS-TX (0.3% Triton X-100 in PBS). All antibodies were dissolved in BB.

#### NeuN+ neurofilament heavy protein+Aβ triple labelling immunohistochemistry

*Day 1:* After BB, sections were incubated overnight at 4°C with a solution of two primary antibodies: a rabbit anti- Neurofilament Heavy Protein antibody (1:200, in BB) and a mouse anti-Aβ antibody (1:200, in BB).

*Day 2: S*ections were incubated with AlexaFluor 635 goat anti-rabbit (1:400, in BB) and then with AlexaFluor 635 goat anti-rabbit (1:400, in BB) plus AlexaFluor 555 donkey anti-mouse (1:400, in BB). Finally, neurons were immunostained using a mouse anti-NeuN antibody conjugated with the fluorochrome AlexaFluor 488 (1:100 in BB).

#### NeuN+ neurofilament heavy protein+GFAP triple labelling immunohistochemistry

*Day 1:* After BB, sections were incubated overnight at 4°C with a solution of two primary antibodies: a rabbit anti- Neurofilament Heavy Protein antibody (1:200, in BB) and a mouse anti-NeuN antibody (1:400, in BB).

*Day 2: S*ections were incubated with AlexaFluor 635 goat anti-rabbit (1:400, in BB) and then with AlexaFluor 635 goat anti-rabbit (1:400, in BB) plus AlexaFluor 555 donkey anti-mouse (1:400, in BB). Finally, astrocytes were immunostained using a mouse anti-GFAP antibody conjugated with the fluorochrome AlexaFluor 488 (1:500 in BB).

#### NeuN+GFAP+IBA1 triple labelling immunohistochemistry

*Day 1*: After BB, sections were incubated overnight at 4°C in a solution with two primary antibodies, a mouse anti-NeuN antibody to immunostain neurons (1:400 in BB) and a rabbit anti-IBA1 antibody to immunostain total microglia (1:300 in BB).

*Day 2*: Sections were incubated for 2 h at room temperature in the dark with AlexaFluor 635 goat anti-rabbit secondary antibody (1:400 in BB) and then for 2 h with a solution of AlexaFluor 635 goat anti-rabbit secondary antibody (1:400 in BB) plus AlexaFluor 555 donkey anti-mouse secondary antibody (1:400 in BB). Finally, astrocytes were immunostained using a mouse anti-GFAP antibody conjugated with the fluorochrome AlexaFluor 488 (1:500 in BB).

#### Aβ+IBA1 double labelling immunohistochemistry

*Day 1*: After BB, sections were incubated overnight at 4°C in a solution containing two primary antibodies: a mouse anti-Aβ antibody (1:200, in BB) plus a rabbit anti-IBA1 antibody (1:300, in BB).

*Day 2*: Sections were incubated with AlexaFluor 488 donkey anti-rabbit (1:400, in BB) and then with a solution of AlexaFluor 488 donkey anti-rabbit (1:400, in BB) plus AlexaFluor 555 donkey anti-mouse (1:400, in BB).

#### NeuN+CD68+IBA1 triple labelling immunohistochemistry

*Day 1*: After BB, sections were incubated overnight at 4°C with a solution containing three primary antibodies: a mouse anti-NeuN antibody (1:400, in BB) plus a rabbit anti-CD68 antibody (1:200, in BB) and a goat anti-IBA1 antibody (1:200, in BB).

*Day 2*: Sections were incubated with AlexaFluor 488 donkey anti-rabbit (1:400, in BB) and then with a solution of AlexaFluor 488 donkey anti-rabbit (1:400, in BB) plus AlexaFluor 635 donkey anti-goat (1:400, in BB). Finally, sections were incubated with a solution of AlexaFluor 488 donkey anti-rabbit (1:400, in BB) plus AlexaFluor 635 donkey anti-goat (1:400, in BB) plus AlexaFluor 555 donkey anti-mouse (1:400, in BB).

#### CX3CR1+IBA1 double labelling immunohistochemistry

*Day 1*: After BB, sections were incubated overnight at 4°C in a solution containing two primary antibodies: a rabbit anti-CX3CR1 antibody (1:200, in BB) plus a goat anti-IBA1 antibody (1:200, in BB).

*Day 2*: Sections were incubated with AlexaFluor 488 donkey anti-rabbit (1:400, in BB) and then with a solution of AlexaFluor 488 donkey anti-rabbit (1:400, in BB) plus AlexaFluor 635 donkey anti-goat (1:400, in BB).

### Microscopy acquisition and images quantitative analyses

Experimenters were blind to treatment during imaging and analysis. All sections were mounted onto gelatin-coated slides using Vectashield mounting medium with DAPI (Vectashield Product Code #H-1200, Vector, Burlingame, CA, United States) and then observed under a LEICA TCS SP8 confocal laser scanning microscope (Leica Microsystems CMS GmbH, Mannheim, Germany) equipped with 20X or 40X objective. Z step was 1.2 µm (20X objective) or 0.6 µm (40X objective). The frequency of acquisition was 200 Hz. and the frame of acquisition was 1024 pixels x 1024 pixels (20X objective) or 2048 pixels x 2048 pixels (40X objective). Confocal scans were acquired keeping all parameters constant.

Confocal acquisitions were performed in the cortical sections cut at stereotaxic coordinate −1700 µm, posterior from Bregma. The regions of interest (ROIs) were the entire cortex, acquired with a ×20 objective as represented in the confocal images, and cortical Layer 5, separately. The ROIs were consistently analyzed in all slices and all quantitative analyses were performed blind by two experimenters. Quantitative analyses were performed on z-projection of 10 consecutive confocal scans (20X objective, z step 1.2 µm, total thickness 12 µm) using the software ImageJ (National Institute of Health, http://rsb.info.nih.gov/ij). For quantitative analyses of immunolabelings, confocal z-projections were converted to TIFF files and thresholded using the Threshold tool of ImageJ. Care was taken to maintain the same threshold in all sections within the same experiment. In each ROI, the pixels above the set threshold were measured and the immunostaining was expressed as positive pixels/total pixels.

Density of Aβ plaques was calculated according to our previous paper (Ugolini et al., 2018). Imunolabeled plaques with a radius >5 μm were identified and counted. Aβ plaque density in the entire cortex and in Layer 5 were calculated separately and expressed as plaques/mm^2^. For the calculation of the density, we measured the area of the ROI (entire cortical thickness or Layer 5, separately) in each confocal image with the tool Measure of ImageJ, and the densities calculated accordingly. Quantitative analyses of Aβ immunolabeling were obtained from the percentage of Aβ positive pixels above a threshold level using the threshold tool of ImageJ (Lana et al., 2024).

Density of neurons immunolabeled with NeuN was expressed as cells/mm^2^. Neuron density in the entire cortex and in Layer 5 (cells/mm^2^) were calculated separately, as written above for the plaques.

Density of astrocytes and microglia were calculated separately, as written above for the plaques, and expressed as cells/mm^2^. We evaluated the density of three populations of microglia, IBA1+ total microglia, CD68^+^ reactive microglia and “ball&chain” phagocytic microglia. The latter are characterized by spherical phagocytic pouches (ball) at the tip of microglial terminal branches (chain), the so-called ball&chain structures (Lana et al., 2023), which can phagocytose apoptotic debris or a small quantity of other substances (Sierra et al., 2010).

To characterize the alteration of the entire cortex and of Layer 5, we measured their thickness with the Measure tool of ImageJ. In each NeuN z-projection image, 3 independent measurements evenly distributed were acquired and averaged (expressed in µm) (Landucci et al., 2022).

Quantitative analyses of GFAP, IBA1, CD68 and CX3CR1 immunofluorescence were obtained from the percentage of Aβ+, GFAP+, IBA1+, CD68^+^ or CX3CR1+ pixels above a threshold level using the threshold tool of ImageJ (for details see (Gerace et al., 2021).

### Statistical analysis

Data are presented as means ± SEM. Statistical significance was evaluated by Student’s t-test, or two-way ANOVA with Genotype and Treatment as the two independent variables followed by Newman-Keuls multiple comparison test, as appropriate. All statistical analyses were performed using GraphPad PRISM v. 8 for Windows (GraphPad Software, San Diego, CA, USA). A probability value (P) of <0.05 was considered significant.

## Results

### The treatment with pre- and probiotics prevents the deposition of Aβ and neuritic plaques in the cortex

**FIGURE 1:**
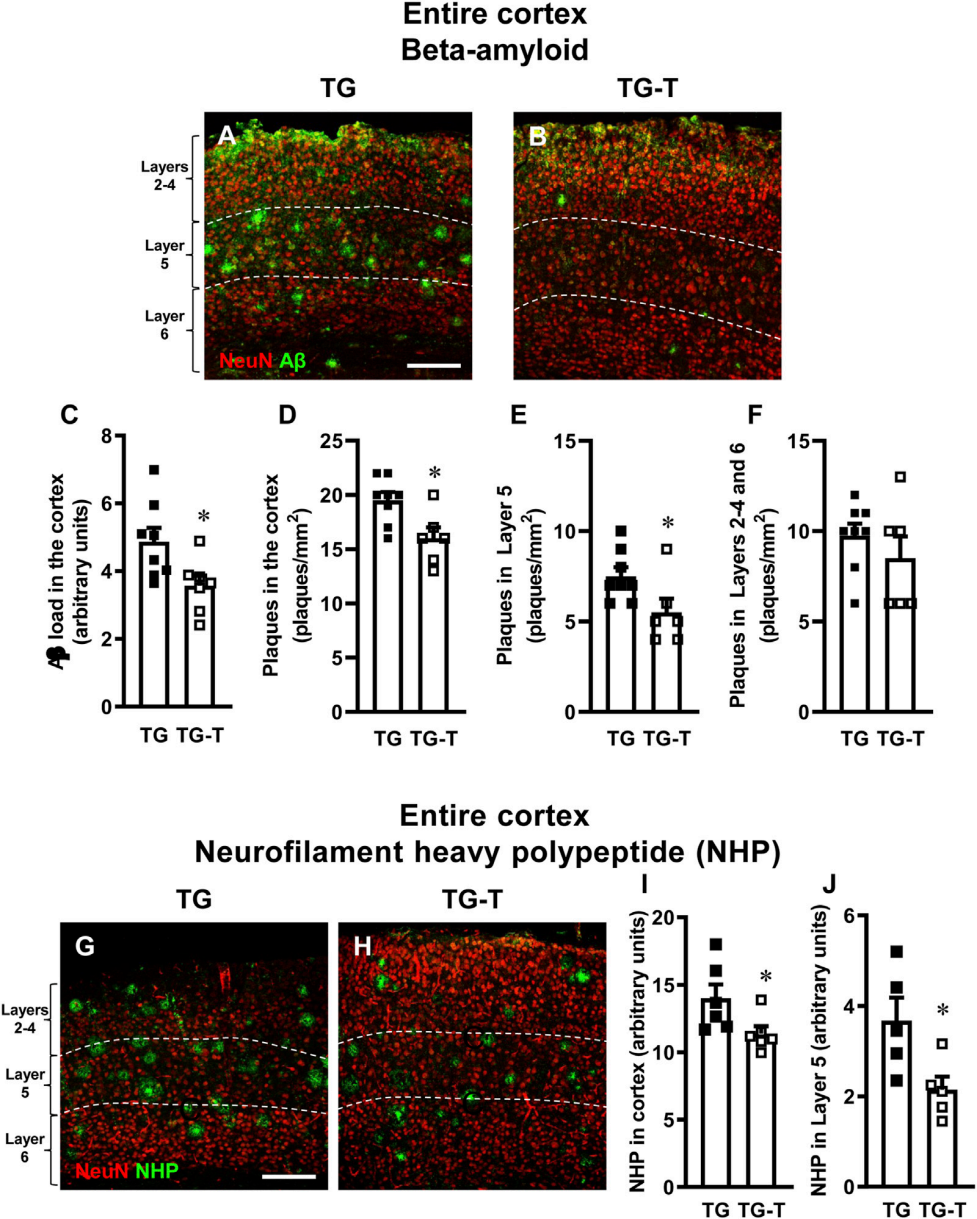
Analysis of Aβ plaques and neurofilament heavy polypeptide (NHP) in the cortex of TG and TG-T mice. **(A,B)** Representative confocal images of Aβ immunostaining (green) in the cortex of a TG (A) and a TG-T (B) mouse. Neurons were immunostained in red. The white dashed lines delineate Layer 5. Scale bar: 100 μm. **(C)** Quantitative analysis of Aβ load (Aβ immunofluorescence) in the cortex of TG (n = 8) and TG-T (n = 6) mice. **(D)** Quantitative analysis of the density of Aβ plaques in the cortex of TG (n = 8) and TG-T (n = 6) mice. **(E)** Quantitative analysis of the density of Aβ plaques in Layer 5 of TG (n = 8) and TG-T (n = 6) mice. **(F)** Quantitative analysis of the density of Aβ plaques in the remaining Layers (2–4 and 6) of TG (n = 8) and TG-T (n = 6) mice. **(G–H)** Representative confocal images of NHP immunostaining (green) in the cortex of TG and TG-T mice. Neurons were immunostained in red. The white dashed lines delineate Layer 5. Scale bar: 100 μm. **(I)** Quantitative analysis of NHP immunofluorescence in the cortex of TG (n = 6) and TG-T (n = 6) mice. **(J)** Quantitative analysis of NHP immunofluorescence in Layer 5 of TG (n = 5) and TG-T (n = 5) mice. Data reported in all graph bars are expressed as mean ± SEM. All statistical analyses were performed by Student’s t-test and significativity was set at P < 0.05.

Aβ and neurons were visualized by immunohistochemistry using anti-Aβ and anti-NeuN antibodies in cortical sections of 8-month-old APP/PS1 mice (Figure 1A, TG) and of APP/PS1 mice fed with pre- and probiotics (Figure 1B, TG-T). NeuN is a specific neuronal marker which is not only localized in the nucleus but also in the cell body cytoplasm. NeuN is a gene product of Rbfox-3, which is a member of the RNA-binding protein Rbfox-1 gene family (Kim et al., 2009). The 46- and 48-kDa subtypes of NeuN/Rbfox3 have distinct subcellular localizations. The 46-kDa subtype is mainly distributed in the nucleus, whereas the 48-kDa subtype is primarily distributed in the cytoplasm (Maxeiner et al., 2014). Thus, anti-NeuN antibody labels not only the nucleus, but also the cytoplasm of neurons.

Qualitative images of the immunolabeled sections are shown in Figures 1A,B, where Aβ peptide is visualized in green, and neurons in red. Quantitative analyses demonstrated that Aβ immunofluorescence, an indication of Aβ deposition in the cortex, was significantly lower in TG-T than in TG mice (−27%, *P < 0.05 TG-T vs. TG, Student’s t test, Figure 1C). Similarly, the density of plaques in the entire cortex was significantly lower in TG-T than in TG mice (−18%, *P < 0.05 TG-T vs. TG, Student’s t test, Figure 1D). The effect of the treatment with pre- and probiotics on Aβ plaques deposition was more efficacious in cortical Layer 5 (−27%, *P < 0.05 TG-T vs. TG, Student’s t test, Figure 1E) than in the cortical layers 2–4 and 6, taken together (−12%, n.s. TG-T vs. TG, Student’s t test, Figure 1F).

Neurofilament heavy polypeptide (NHP), present in neurofibrillary tangles and degenerating plaque neurites (pathogenic markers of AD) and neurons were visualized by immunohistochemistry with selective antibodies. Qualitative images in Figures 1G,H (NHP in green, neurons in red) show that NHP is present in the cortex of APP/PS1 mice, especially in Layer 5. The quantitative analyses of NHP immunofluorescence (Figures 1I,J) show that the treatment with pre- and probiotics decreased significantly the presence of degenerating plaque neurites in the entire cortex of TG-T mice (−19%, *P < 0.05 TG-T vs. TG, Student’s t test, Figure 1I). This effect was most pronounced in Layer 5 (−41%, *P < 0.05 TG-T vs. TG, Student’s t test, Figure 1J).

**FIGURE 2:**
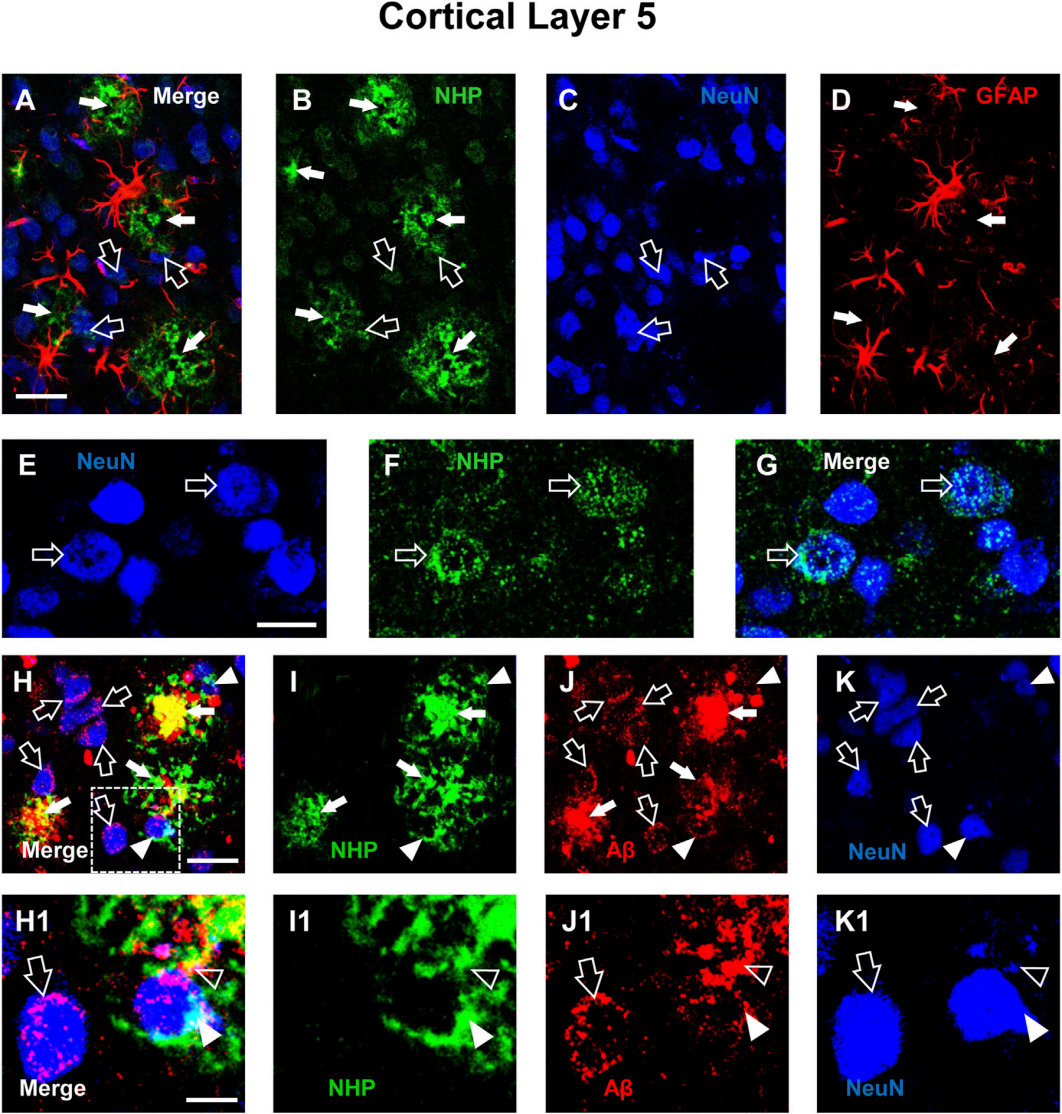
Qualitative analysis of the interplay among neurons, astrocytes, neurofibrillary tangles (NHP) and Aβ deposits in the cortex of a TG mouse. **(A–D)** Representative confocal images of triple labelling immunostaining of NHP (B, green), neurons (C, blue), and astrocytes (D, red) in the cortex of a TG mouse. A is the merge of the three fluorescence channels. Each image is the z-projection of 10 consecutive confocal scans acquired with a 20x objective, each 1.2 µm thick (total thickness 12 µm). Open arrows indicate NHP localized within neurons, white arrows NHP in the parenchyma. Scale bar: 25 µm. **(E–G)** Representative confocal images of double labelling immunostaining of NeuN (E, blue) and NHP (F, green). G is the merge of the two fluorescence channels. Each image is the z-projection of 4 consecutive confocal scan acquired with a 40x objective, each 0.6 µm thick (total thickness 2.4 µm). Open arrows indicate NHP localized within neurons. Scale bar: 10 µm. **(H–K)** Representative confocal images of triple labelling immunostaining of NHP (I, green), Aβ (J, red), and neurons (K, blue) in the cortex of a TG mouse. H is the merge of the three fluorescence channels. Each image is the z-projection of 5 consecutive confocal scans acquired with a 40x objective, each 0.6 µm thick (total thickness 3 µm). Open arrows indicate Aβ localized within neurons, white arrows neuritic plaques in the parenchyma, arrowheads NHP and Aβ in neuron cytoplasm. Scale bar: 20 μm. **(H1–K1)** Enlargement of the framed areas of the above images. Each image is one single confocal scan acquired with a 40x objective, total thickness 0.6 µm. Open arrows indicate Aβ localized within neurons, white arrows neuritic plaques in the parenchyma, arrowheads NHP and Aβ in neuron cytoplasm. Scale bar: 5 µm.

We performed qualitative analyses of the distribution of NHP in cortical Layer 5 of TG and TG-T mice. Figures 2A–D show the triple immunostaining of NHP (green), neurons (blue), and astrocytes (red). Figure 2A, a merge of the 3 channels of fluorescence shown in Figures 2B–D, is the z-projection of 2 consecutive confocal scans taken with a ×40 objective (0.6 µm each, total thickness 1.2 µm). From the images it is possible to appreciate that NHP immunostaining is present in cortical parenchyma where it is aggregated to form many neuritic plaques (white arrows, Figures 2A,B,D). Hypertrophic astrocytes are arranged around neuritic plaques, evidenced by the white arrows in Figure 2D. It appears that NHP immunostaining is also present in the cytoplasm of many degenerating neurons (open arrows, Figures 2A–C). The localization of NHP in the cytoplasm of degenerating neurons is more clearly visible in the images reported in Figures 2E–G, which show z-projections of 4 confocal scans (0.6 µm each, total thickness 2.4 µm), of neurons (blue, Figure 2E), NHP (green, Figure 2F), and the merge of the 2 previous images. It is evident that NHP was highly expressed in the cytoplasm of the 2 neurons, as indicated by the open arrows. The nucleus of the left neuron is fragmented, an index that the neuron is in degeneration. Neuritic plaques were characterized using triple immunostaining of NHP (Figure 2I, green), Aβ (Figure 2J red), and neurons (Figure 2K, blue). Figure 2H, a merge of the 3 channels of fluorescence shown in Figures 2I–K, is the z-projection of 5 consecutive confocal scans taken with a ×40 objective (0.6 µm each, total thickness 3.0 µm). It is evident from the images (Figures 2H–J), that NHP aggregated with Aβ to form neuritic plaques (orange-yellow color), surrounded by astrocytes (as evidenced in Figure 2A). The core of the plaques was formed by NHP that colocalized with Aβ (yellow-orange color) while in the more external part, NHP (green) was juxtaposed, not overlapped, to Aβ (red). Many neurons surrounding and in the vicinity of the neuritic plaques (open arrows) showed Aβ aggregates in their cytoplasm, mainly in the cytoplasm close to the plasma membrane. It is interesting to note that two neurons (indicated by the white arrowheads), in close contact with two neuritic plaques, had both NHP (Figure 2I) and Aβ (Figure 2J) aggregates in their cytoplasm. The enlargements of the framed area of Figure 2H, shown in Figures 2H1–K1 show different phenomic states of 3 neurons in the surroundings of the neuritic plaque (the images are enlargements of 1 single confocal scan taken with a 40X objective, total thickness 0.6 µm). The open arrows show neurons with aggregates of Aβ in the cytoplasm, mainly close to the plasma membrane. The white arrowheads show a neuron in close contact with the neuritic plaque containing aggregates of NHP and Aβ (the colocalization is indicated by the cyan-white color in the merged image, Figure 2H1). Fragments of a neuron (indicated by the open arrowheads), colocalize with aggregates of NHP and Aβ and are incorporated in the neuritic plaque. The symbiotic treatment with pre- and probiotics decreased the formation of neuritic plaques, as shown in the quantitative analyses of Figures 1I,J.

### The treatment with pre- and probiotics prevents neurodegeneration in the cortex

**FIGURE 3:**
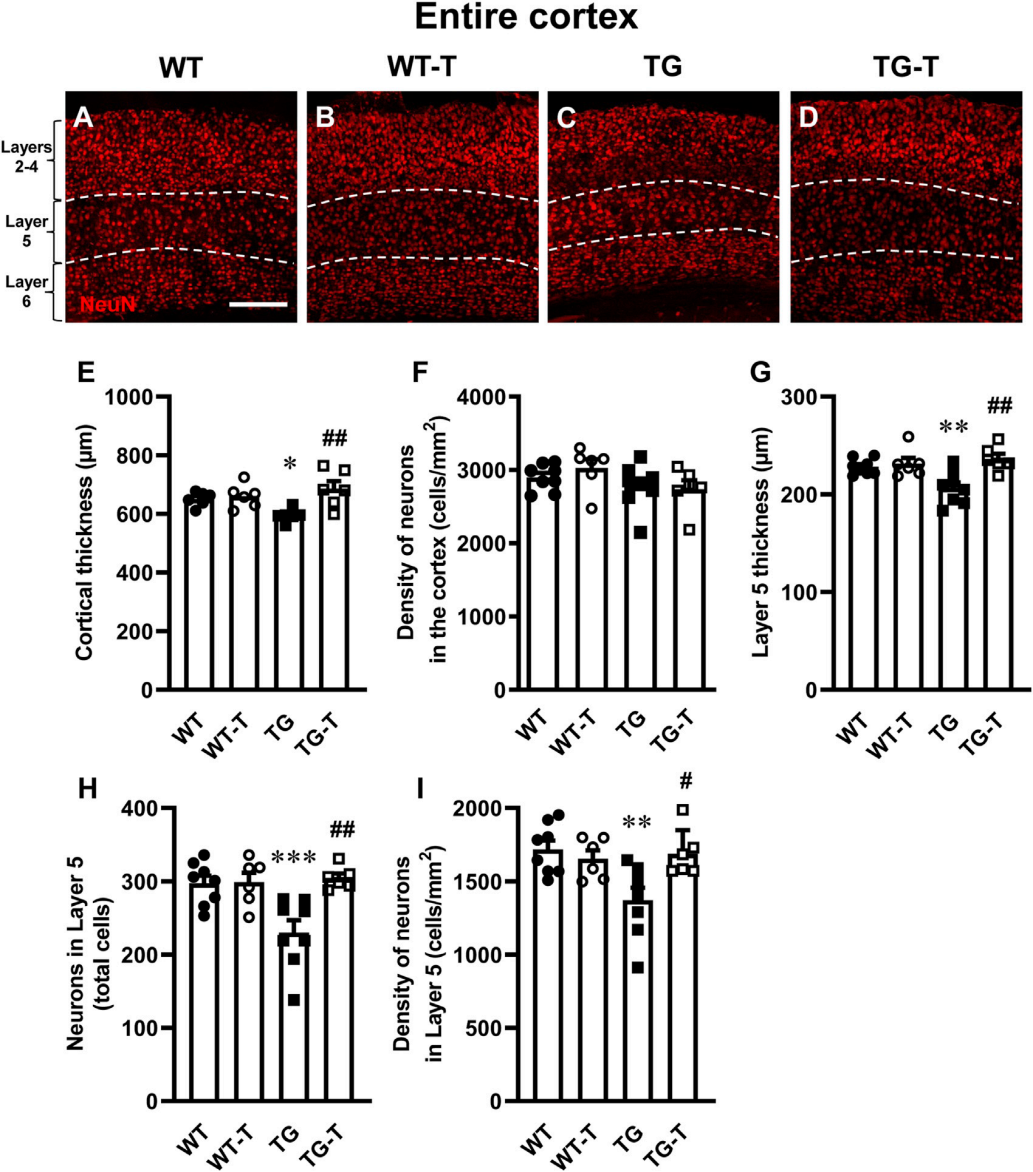
Analysis of neurons in the cortex of WT, WT-T, TG and TG-T mice. **(A–D)** Representative confocal images of NeuN immunostaining of neurons (red) in the cortex of a WT **(A)**, a WT-T **(B)**, a TG **(C)**, and a TG-T mouse **(D)**. The white dashed lines delineate Layer 5. Scale bar: 100 μm. **(E)** Quantitative analysis of the cortical thickness of WT (n = 6), WT-T (n = 6), TG (n = 6), and TG-T (n = 6) mice. **(F)** Quantitative analysis of neuronal density (neurons/mm^2^) in the cortex of WT (n = 8), WT-T (n = 6), TG (n = 8), and TG-T (n = 6) mice. **(G)** Quantitative analysis of the thickness of Layer 5 of WT (n = 8), WT-T (n = 6), TG (n = 8), and TG-T (n = 6) mice. **(H)** Quantitative analysis of total neurons in Layer 5 of WT (n = 8), WT-T (n = 6), TG (n = 8), and TG-T (n = 6) mice. **(I)** Quantitative analysis of neurons/mm^2^ in Layer 5 of WT (n = 8), WT-T (n = 6), TG (n = 8), and TG-T (n = 6) mice. Data reported in all graph bars are expressed as mean ± SEM. All statistical analyses were performed by two way ANOVA followed by Newman-Keuls multiple comparison test and significativity was set at P < 0.05.

Using immunohistochemical staining with anti-NeuN antibody, we evaluated the thickness of the cortex from Layer 2 to Layer 6 and the density of neurons (Figure 3 red) in cortical sections of wild type mice (WT, Figure 3A), wild type mice fed with probiotics and prebiotics (WT-T, Figure 3B), APP/PS1 mice (TG, Figure 3C) and APP/PS1 mice fed with pre- and probiotics (TG-T, Figure 3D). Qualitative images of immunolabelled cortical sections are shown in Figures 3A–D. The graph in Figure 3E, shows the quantitative analysis of the entire cortical thickness, and demonstrates a small but significant shrinkage of the cortex in TG mice (−9% vs. WT mice; Statistical analysis: two-way ANOVA: Genotype F_(1,20)_ = 0.8619, n.s.; Treatment F_(1,20)_ = 9.190, P < 0.01, Interaction F_(1,20)_ = 5.849, P < 0.05; Newman-Keuls multiple comparison test: *P < 0.05 TG vs. WT; ^##^P < 0.01 TG-T vs. TG). However, the density of neurons in the entire thickness of the cortex of TG mice, although slightly lower than those in WT mice (−6%, n.s.) was not statistically different among the four experimental groups (Statistical analysis: two-way ANOVA showed no significant differences among groups), as shown in Figure 3F.

Taking into consideration the results of the quantitative analyses of Aβ plaques shown in Figure 1 and in the previous paragraph, we performed a separate quantitative analysis of the thickness and of the density of neurons of cortical Layer 5, which had been previously shown to be the most damaged layer in AD models (Chen et al., 2022). The graph in Figure 3G demonstrates a significant shrinkage of Layer 5 in TG mice (−10% vs. WT mice). This effect was significantly attenuated by the treatment with pre- and probiotics (Statistical analysis: two-way ANOVA: Genotype F_(1,24)_ = 2.780, n.s.; Treatment F_(1,24)_ = 10.660, P < 0.01, Interaction F_(1,24)_ = 6.938, P < 0.05; Newman-Keuls multiple comparison test: **P < 0.01 TG vs. WT; ^##^P < 0.01 TG-T vs. TG). The quantitative analyses of neurons showed that both the total number, and the density of neurons decreased significantly in Layer 5 of TG mice, and the treatment significantly prevented this loss (Figures 3H,I, respectively). Figure 3H shows that the significant decrease of neurons (−23% TG vs. WT) in cortical Layer 5 of TG mice was significantly attenuated by the treatment (Statistical analysis: two-way ANOVA: Genotype F_(1,24)_ = 5.560, P < 0.05; Treatment F_(1,24)_ = 8.468, P < 0.01, Interaction F_(1,24)_ = 7.812, P < 0.05; Newman-Keuls multiple comparison test: ***P < 0.001 TG vs. WT; ^##^P < 0.01 TG-T vs. TG). Figure 3I shows that neuronal density in cortical Layer 5 of TG mice decreased significantly (−20% TG vs. WT). The treatment significantly prevented this effect (Statistical analysis: two-way ANOVA: Genotype F_(1,24)_ = 4.856, P < 0.05; Treatment F_(1,24)_ = 3.178, n.s., Interaction F_(1,24)_ = 7.112, P < 0.05; Newman-Keuls multiple comparison test: **P < 0.01 TG vs. WT; ^#^P < 0.05 TG-T vs. TG). The neuroprotection caused by the treatment in cortical Layer 5 matches with the above-described decrease of neuritic plaques (see Figure 1).

### Characterization of astrocytes in cortex

**FIGURE 4:**
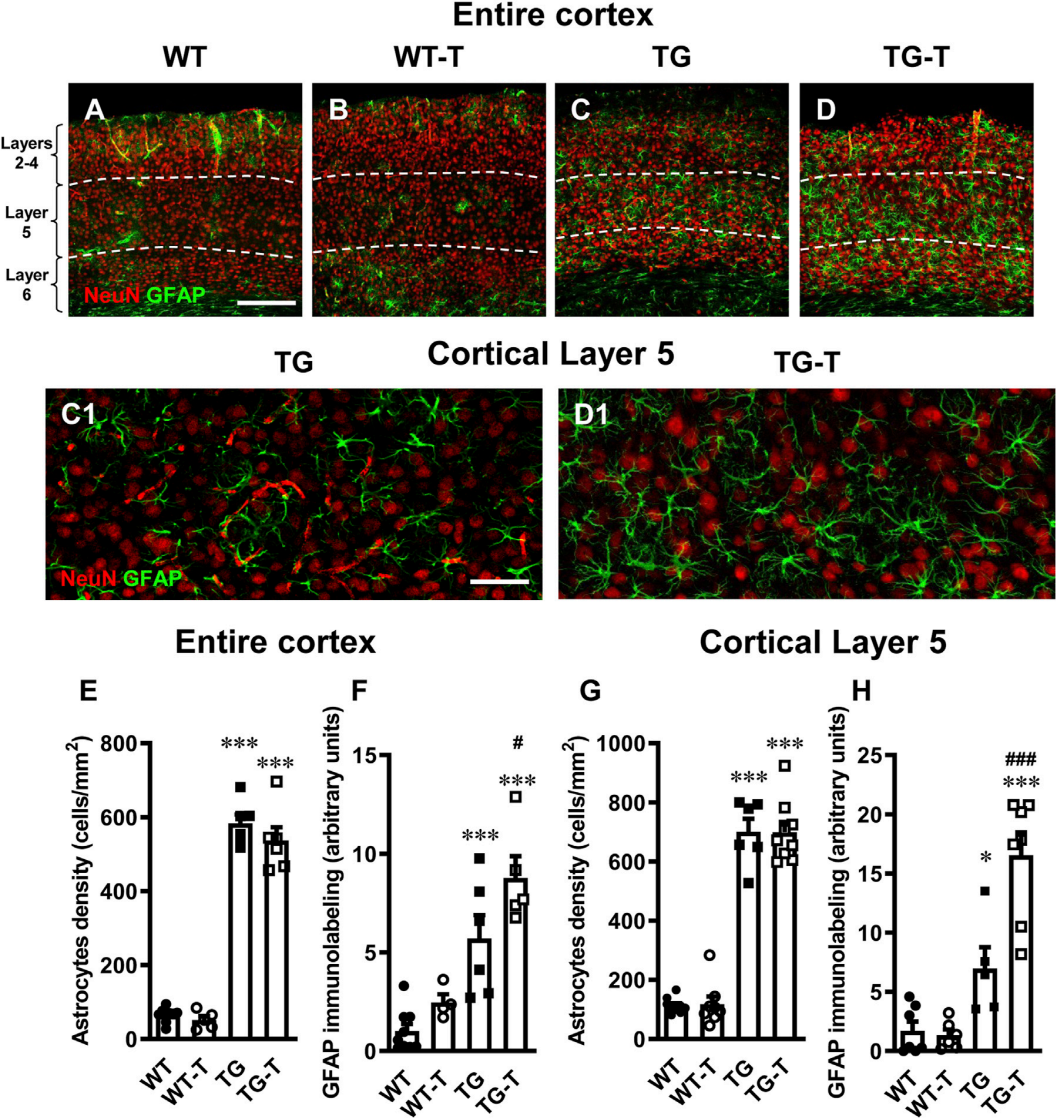
Analysis of astrocytes in the cortex of WT, WT-T, TG and TG-T mice. **(A–D)** Representative confocal images of GFAP immunostaining of astrocytes (green) and neurons (red) in the cortex of a WT **(A)**, a WT-T **(B)**, a TG **(C)** and a TG-T **(D)** mouse. The white dashed lines delineate Layer 5. Scale bar: 100 μm **(A–D)**. **(C1, D1)**: Enlargements of Layer 5 of the above images in C and D. Scale bar: 40 µm. **(E)** Quantitative analysis of GFAP+ astrocyte density (cells/mm^2^) in the cortex of WT (n = 9), WT-T (n = 5), TG (n = 6), and TG-T (n = 6) mice. **(F)** Quantitative analysis of GFAP immunofluorescence in the cortex of WT (n = 9), WT-T (n = 5), TG (n = 6), and TG-T (n = 5) mice. **(G)** Quantitative analysis of GFAP+ astrocyte density (cells/mm^2^) in Layer 5 of WT (n = 10), WT-T (n = 8), TG (n = 6), and TG-T (n = 9) mice. **(H)** Quantitative analysis of GFAP immunofluorescence in Layer 5 of WT (n = 8), WT-T (n = 6), TG (n = 6), and TG-T (n = 7) mice. Data reported in all graph bars are expressed as mean ± SEM. All statistical analyses were performed by two way ANOVA followed by Newman-Keuls multiple comparison test and significativity was set at P < 0.05.

Astrocytes were visualized by immunohistochemistry with anti-GFAP antibody (Figures 4A–D1, green) in cortex sections of wild type mice (WT, Figure 4A), wild type mice fed with probiotics and prebiotics (WT-T, Figure 4B), APP/PS1 mice (TG, Figure 4C), and APP/PS1 mice treated with pre- and probiotics (TG-T, Figure 4D). GFAP (glial fibrillary acidic protein) is a widely used marker of astrocytes (Yang and Wang, 2015). Neurons were immunostained with anti NeuN antibody (Figures 4A–D1, red). Enlargements of Layer 5 of TG and TG-T mice are shown in Figures 4C1–D1. From the representative confocal images, it is apparent that astrocytes are more numerous and more activated in the cortex of both TG and TG-T mice in comparison to WT and WT-T mice. It is possible to envisage that astrocytes activation is mainly around plaques (see also Figures 2A–D).

The graphs in Figures 4E,F show the results of quantitative analyses of astrocytes density and GFAP expression in the entire cortex of the four experimental groups. There was no significant difference in astrocyte density in the cortex of WT and WT-T mice. Astrocytes density increased significantly in TG (+ 888% TG vs. WT) and TG-T mice (+ 810% TG vs. WT), compared to WT and WT-T mice (Figure 4E) (Statistical analysis: two-way ANOVA: Genotype F_(1,22)_ = 572.0, P < 0.0001; Treatment F_(1,22)_ = 2.022, n.s., Interaction F_(1,22)_ = 0.5879, n.s.; Newman-Keuls multiple comparison test: ***P < 0.001 TG vs. WT, and TG-T vs. WT; TG-T vs. TG, n.s.).

In the cortex of WT treated mice, we observed a slight, not significant, increase in the expression of GFAP, in comparison to WT mice. In the cortex of TG mice, the expression of GFAP was significantly higher than in WT and WT-T mice (+433%, TG vs. WT), and it was further increased by the treatment (+744, TG-T vs. WT; +54% TG-T vs. TG, Figure 4F) (Statistical analysis: two-way ANOVA: Genotype F_(1,20)_ = 42.95, P < 0.001; Treatment F_(1,20)_ = 7.307, P < 0.05, Interaction F_(1,20)_ = 0.9335, n.s.; Newman-Keuls multiple comparison test: ***P < 0.001 TG vs. WT, and TG-T vs. WT; ^#^P < 0.05 TG-T vs. TG).

The graphs in Figures 4G,H show the results of astrocytes density and GFAP expression in Layer 5 of the four experimental groups, respectively. There was no significant difference in astrocyte density in Layer 5 of WT and WT-T mice. Astrocytes density increased significantly in TG (+ 579% TG vs. WT) and TG-T mice (+ 576% TG vs. WT), compared to WT and WT-T mice (Figure 4G) (Statistical analysis: two-way ANOVA: Genotype F_(1,28)_ = 399.3, P < 0.001; Treatment F_(1,28)_ = 0.00467, n.s., Interaction F_(1,28)_ = 0.0135, n.s.; Newman-Keuls multiple comparison test: ***P < 0.001 TG vs. WT, and TG-T vs. WT; TG-T vs. TG n.s.).

In cortical Layer 5 of TG mice, the expression of GFAP was significantly higher than in WT and WT-T mice (+302%, TG vs. WT), and it was further increased by the treatment (+853%, TG-T vs. WT; +138% TG-T vs. TG, Figure 4H) (Statistical analysis: two-way ANOVA: Genotype F_(1,21)_ = 52.69, P < 0.001; Treatment F_(1,21)_ = 10.63, P < 0.01, Interaction F_(1,21)_ = 12.53, P < 0.01; Newman-Keuls multiple comparison test: *P < 0.05 TG vs. WT, ***P < 0.001 TG-T vs. WT; ^###^P < 0.001 TG-T vs. TG).

### Characterization of microglia in the cortex

**FIGURE 5:**
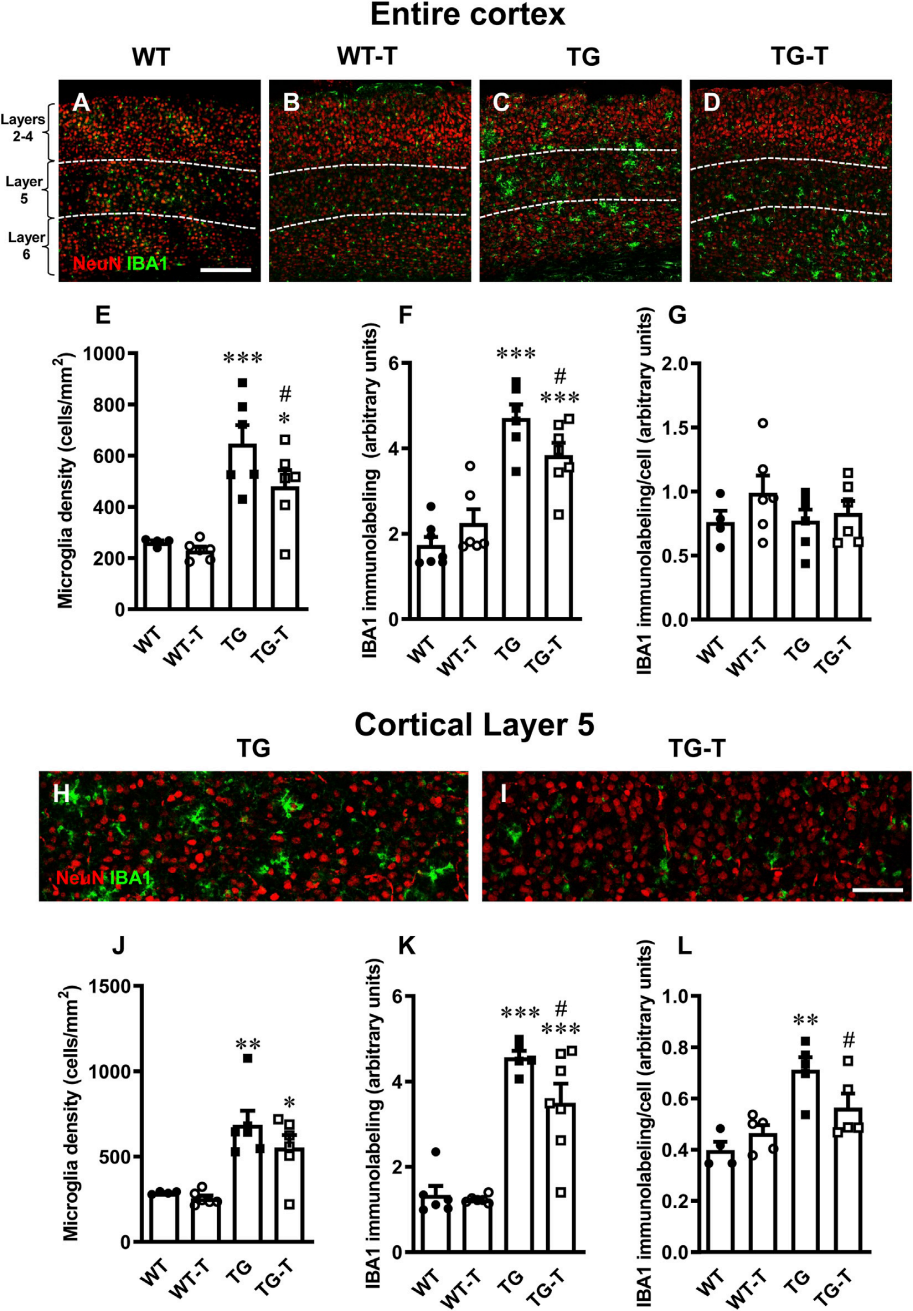
Analysis of total microglia in the cortex of WT, WT-T, TG and TG-T mice. **(A–D)** Representative confocal images of IBA1 immunostaining of microglia (green) and neurons (red) in the cortex of WT, WT-T, TG and TG-T mice. The white dashed lines delineate Layer 5. Scale bar: 100 μm. **(E)** Quantitative analysis of IBA1+ total microglia density in the cortex of WT (n = 4), WT-T (n = 6), TG (n = 6), and TG-T (n = 6) mice (cells/mm^2^). **(F)** Quantitative analysis of IBA1 immunofluorescence in the cortex of WT (n = 7), WT-T (n = 6), TG (n = 6), and TG-T (n = 7) mice. **(G)** Quantitative analysis of IBA1 immunofluorescence per microglia (% positive pixels/cell) in the cortex of WT (n = 4), WT-T (n = 6), TG (n = 6), and TG-T (n = 6) mice. **(H,I)** Representative confocal images of IBA1 immunostaining of microglia (green) and neurons (red) in Layer 5 of TG and TG-T mice. Scale bar: 50 μm. **(J)** Quantitative analysis of IBA1+ total microglia density in Layer 5 of WT (n = 4), WT-T (n = 6), TG (n = 6), and TG-T (n = 6) mice (cells/mm^2^). **(K)** Quantitative analysis of IBA1 immunofluorescence in Layer 5 of WT (n = 6), WT-T (n = 6), TG (n = 5), and TG-T (n = 7) mice. **(L)** Quantitative analysis of IBA1 immunofluorescence per microglia (% positive pixels/cell) in Layer 5 of WT (n = 4), WT-T (n = 5), TG (n = 5), and TG-T (n = 5) mice. Data reported in all graph bars are expressed as mean ± SEM. All statistical analyses were performed by two way ANOVA followed by Newman-Keuls multiple comparison test and significativity was set at P < 0.05.

**FIGURE 6:**
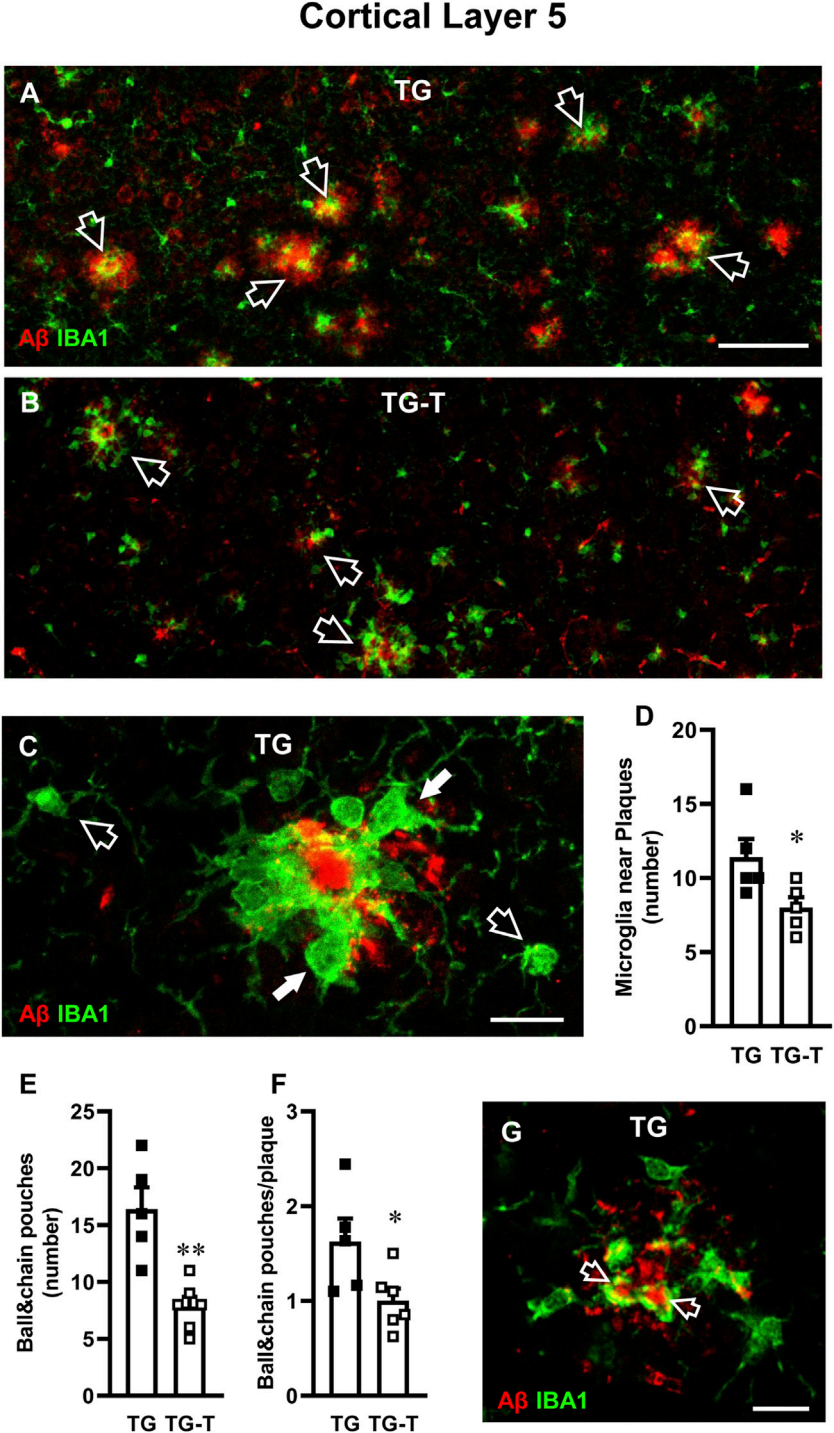
Analysis of ball&chain phagocytic microglia in Layer 5 of TG and TG-T mice. **(A,B)** Representative confocal images of immunostaining of microglia (IBA1, green) and Aβ plaques (red) in Layer 5 of TG and TG-T mice. Each image is the z-projection of 10 consecutive confocal scan acquired with a ×20 objective, each 1.2 µm thick (total thickness 12 µm). Open arrows indicate Aβ plaques surrounded by ball&chain phagocytic microglia. Scale bar: 50 μm. **(C)** Representative confocal image (acquired with ×40 objective) of microglia (IBA1, green) and Aβ (red) in Layer 5 The image is the z-projection of 10 confocal scans (each 0.6 μm, total thickness 6 µm). Microglia around amyloid plaques (white arrows) and two microglia cells far from plaques (open arrows) are pointed out. Scale bar: 10 μm. **(D)** Quantitative analysis of microglia near plaques in Layer 5 of TG (n = 5) and TG-T mice (n = 5). **(E)** Quantitative analysis of ball&chain pouches in Layer 5 of TG (n = 5) and TG-T mice (n = 6). **(F)** Quantitative analysis of ball&chain pouches/plaque in Layer 5 of TG (n = 5) and TG-T mice (n = 6). **(G)** Representative confocal image (acquired with ×40 objective) of microglia (IBA1, green) and Aβ (red) in Layer 5 of a TG mouse to evidence the engulfment of Aβ by ball&chain phagocytic microglia pouches (open arrows). The image is the z-projection of a single confocal scan (total thickness 0.6 µm). Scale bar: 10 μm. Data reported in all graph bars are expressed as mean ± SEM. All statistical analyses were performed by Student’s t-test and significativity was set at P < 0.05.

To perform the quantitative analysis of total microglia in cortical sections of wild type mice (WT), wild type mice fed with pre- and probiotics (TG-T), APP/PS1 mice (TG) and APP/PS1 mice fed with pre- and probiotics (TG-T), we immunolabelled microglia with anti-IBA1 antibody (Figures 5A–D,H,I, green), and neurons with anti NeuN antibody (Figures 5A–D,H,I, red). Images of fluorescent immunostaining show that microglia changed their morphological features in the cortex (Figures 5, 6), especially around amyloid plaques. The Ionized calcium binding adaptor molecule 1 (IBA1) is one of the most well-established markers of microglia.

Quantitative analysis showed that microglia density, total IBA1 expression and IBA1 expression/cell in the cortex of WT mice were not different from those of WT-T mice (Figures 5D,E). The density of microglia increased significantly in the cortex of TG, compared to WT mice (Figure 5E). However, in TG-T mice, the density of cortical microglia was significantly lower than in TG mice (−26% TG-T vs. TG, Figure 5E) (Statistical analysis: two-way ANOVA: Genotype F_(1,18)_ = 34.00, P < 0.001; Treatment F_(1,18)_ = 3.337, n.s., Interaction F_(1,18)_ = 1.505, n.s.; Newman-Keuls multiple comparison test: ***P < 0.001 TG vs. WT, *P < 0.05 TG-T vs. WT; ^#^P < 0.05 TG-T vs. TG).

The expression of IBA1 was significantly higher in the cortex of TG mice in comparison to WT and WT-T mice, but in the cortex of TG-T mice the treatment decreased significantly the increase of IBA1 expression (−19%, TG-T vs. TG, Figure 5F) (Statistical analysis: two-way ANOVA: Genotype F_(1,22)_ = 65.30, P < 0.001; Treatment F_(1,22)_ = 0.4014, n.s., Interaction F_(1,22)_ = 5.982, P < 0.05; Newman-Keuls multiple comparison test: ***P < 0.001 TG vs. WT, ***P < 0.001 TG-T vs. WT; ^#^P < 0.05 TG-T vs. TG). However, the expression of IBA1/cell did not change significantly in TG or TG-T mice, compared to WT and WT-T mice (Figure 5G) (Statistical analysis: two-way ANOVA showed no significant differences among groups.)

We performed the analysis of microglia in Layer 5 of the cortex, see the qualitative images of TG and TG-T mice in Figures 5H,I. We found a highly significant increase in microglia density in Layer 5 of TG mice (Figure 5J). The treatment caused a non-significant decrease in microglia density (−20%, TG-T vs. TG, Figure 5J) (Statistical analysis: two-way ANOVA: Genotype F_(1,18)_ = 31.20, P < 0.001; Treatment F_(1,18)_ = 1.792, n.s., Interaction F_(1,18)_ = 0.6568, n.s.; Newman-Keuls multiple comparison test: **P < 0.01 TG vs. WT, *P < 0.05 TG-T vs. WT; TG-T vs. TG, n.s.). Nevertheless, IBA1 expression in Layer 5 of TG mice was higher than in WT and WT-T mice, and in the cortex of TG-T mice the treatment with pre- and probiotics attenuated significantly the increase of IBA1 expression (−18%, TG-T vs. TG) (Figure 5K) (Statistical analysis: two-way ANOVA: Genotype F_(1,20)_ = 85.25, P < 0.001; Treatment F_(1,20)_ = 3.870, n.s., Interaction F_(1,20)_ = 2.625, n.s.; Newman-Keuls multiple comparison test: ***P < 0.001 TG vs. WT, ***P < 0.001 TG-T vs. WT; ^#^P < 0.05 TG-T vs. TG).

Interestingly, in Layer 5 of TG mice, the expression of IBA1/cell was significantly higher than in WT and WT-T mice, indicating that in TG mice each microglia cell was bigger and possibly more activated than in WT and WT-T mice. The treatment with pre- and probiotics prevented significantly microglia activation (−21%, TG-T vs. TG, Figure 5L) (Statistical analysis: two-way ANOVA: Genotype F_(1,15)_ = 21.76, P < 0.001; Treatment F_(1,15)_ = 0.837, n.s., Interaction F_(1,15)_ = 5.855, P < 0.05; Newman-Keuls multiple comparison test: **P < 0.01 TG vs. WT; ^#^P < 0.05 TG-T vs. TG). The treatment with pre-and probiotics attenuated microglia activation in Layer 5 of the cortex.

In Layer 5 of TG mice, activated microglia was mainly located around amyloid plaques, as shown by the qualitative image presented in Figure 6A, which shows the immunolabelling of microglia (green) and Aβ (red). Figure 6C shows the different morphological features of microglia located close (white arrows) or far (open arrows) from an amyloid plaque in Layer 5 of a TG mouse. In TG-T mice, the distribution of microglia around plaques was significantly lower than in TG mice (−30% TG-T vs. TG, Statistical analysis: Student’s t test: *P < 0.05 TG-T vs. TG), as shown in Figure 6D.

We evaluated “ball&chain” phagocytic microglia, characterized by spherical phagocytic pouches (ball) at the tip of microglial terminal branches (chain), which can phagocytose apoptotic debris or Aβ plaques (Sierra et al., 2010). Most ball&chain microglia in Layer 5 of TG mice surrounded amyloid plaques (Figures 6A,B).

We quantified the spherical phagocytic pouches of ball&chain microglia in Layer 5 and we found that the treatment with pre- and probiotics significantly decreased both total pouches (−52%, TG-T vs. TG, Statistical analysis: **P < 0.01 TG-T vs. TG, Student’s t test; Figure 6E) and the pouches located around plaques (−38% TG-T vs. TG, Statistical analysis: *P < 0.05 TG-T vs. TG, Student’s t test; Figure 6F). The ball&chain microglia pouches located around plaques were actively phagocytosing Aβ, as shown by the orange-yellow color evidenced by the open arrows in the qualitative double confocal image shown in Figure 6G. The image shows a z-projection of a single confocal scan (total thickness 0.6 µm), merge of IBA1 (green), and Aβ immunolabelling (red).

**FIGURE 7:**
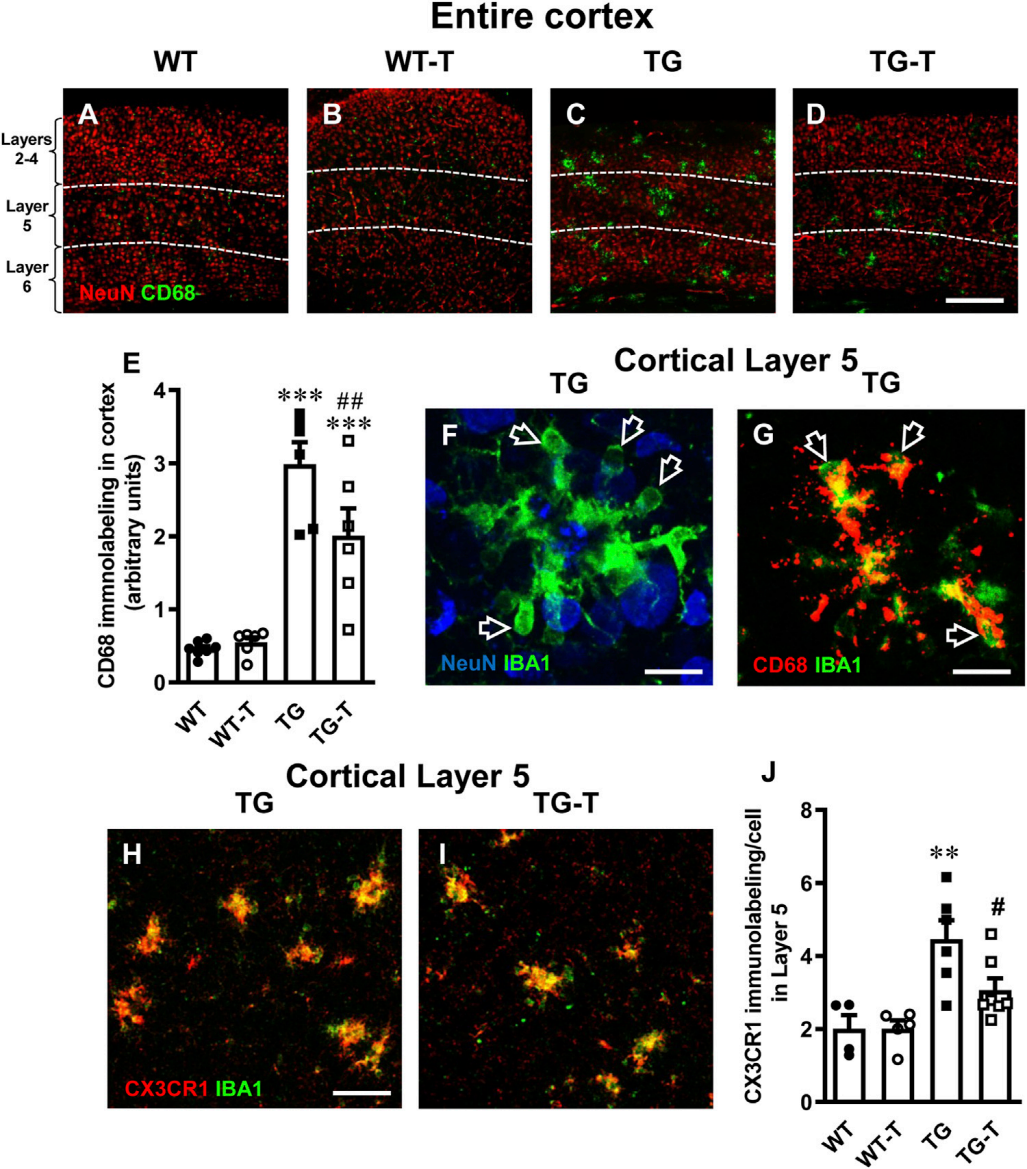
Analysis of activated microglia in the cortex of WT, WT-T, TG and TG-T mice. **(A–D)** Representative confocal images of CD68 immunostaining of microglia (green) and neurons (red) in the cortex of WT, WT-T, TG and TG-T mice. The white dashed lines delineate Layer 5. Scale bar: 100 μm. **(E)** Quantitative analysis of CD68^+^ activated microglia density in the cortex of WT (n = 7), WT-T (n = 6), TG (n = 6), and TG-T (n = 6) mice (cells/mm^2^). **(F)** Representative confocal image of IBA1 immunostaining of microglia (green) and neurons (blue) in Layer 5 of a TG mouse. Total thickness 21 µm. Scale bar: 10 μm. (**F)** Representative confocal image of IBA1 immunostaining of microglia (green) and CD68 (red) in Layer 5 of a TG mouse. Total thickness 6 µm. Scale bar: 10 μm. **(H,I)** Representative confocal images of IBA1 immunostaining of microglia (green) and CX3CR1 (red) in Layer 5 of a TG and a TG-T mouse. Scale bar: 10 μm. **(J)** Quantitative analysis of CX3CR1 immunofluorescence in Layer 5 of WT (n = 4), WT-T (n = 5), TG (n = 6), and TG-T (n = 7) mice. Data reported in all graph bars are expressed as mean ± SEM. All statistical analyses were performed by two way ANOVA followed by Newman-Keuls multiple comparison test and significativity was set at P < 0.05.

To further evidence the activation of microglia, we performed immunostaining with anti CD68 antibody (Figures 7A–D, green) and neurons were immunostained with NeuN antibody (Figures 7A–D, red). Cluster of Differentiation 68 is a marker of the phagocytic phenotype of microglia. Quantitative analysis of microglia activation in the cortex, defined by CD68 immunofluorescence expression, is shown in Figure 7E. In TG mice, CD68 expression was significantly higher than in WT and WT-T mice, and was decreased significantly by the treatment (−33%, TG-T vs. TG, Figure 7E) (Statistical analysis: two-way ANOVA: Genotype F_(1,21)_ = 71.79, P < 0.001; Treatment F_(1,21)_ = 3.581, n.s., Interaction F_(1,21)_ = 5.143, P < 0.05; Newman-Keuls multiple comparison test: ***P < 0.001 TG vs. WT; ***P < 0.001 TG-T vs. WT; ^##^P < 0.01 TG-T vs. TG). Figure 7F shows an enlargement of ball&chain microglia that highlights its morphology, with the open arrows that indicate the ball&chain pouches. Double labelling of microglia with IBA1 (green) and CD68 (red), shown in Figure 7G, demonstrate that ball&chain pouches expressed CD68, a marker of microglia activation (yellow-orange color, open arrows).

Fraktalkine receptor CX3CR1 is expressed on microglia cells and is involved in their phagocytic activity, since its ligand fraktalkine acts as a chemokine and drives microglia towards the targets of phagocytosis. Analyzing the fractalkine receptor CX3CR1, we found that it was highly expressed in microglia of TG mice (+122% TG vs. WT, Figure 7H). The quantitative analysis revealed that the treatment with pre- and probiotics decreased significantly CX3CR1 expression (−33%, TG-T vs. TG, Figure 7J) (Statistical analysis: two-way ANOVA: Genotype F_(1,18)_ = 19.45, P < 0.001; Treatment F_(1,18)_ = 3.049, n.s., Interaction F_(1,18)_ = 3.049, n.s.; Newman-Keuls multiple comparison test: **P < 0.01 TG vs. WT; ^#^P < 0.05 TG-T vs. TG).

## Discussion

**FIGURE 8:**
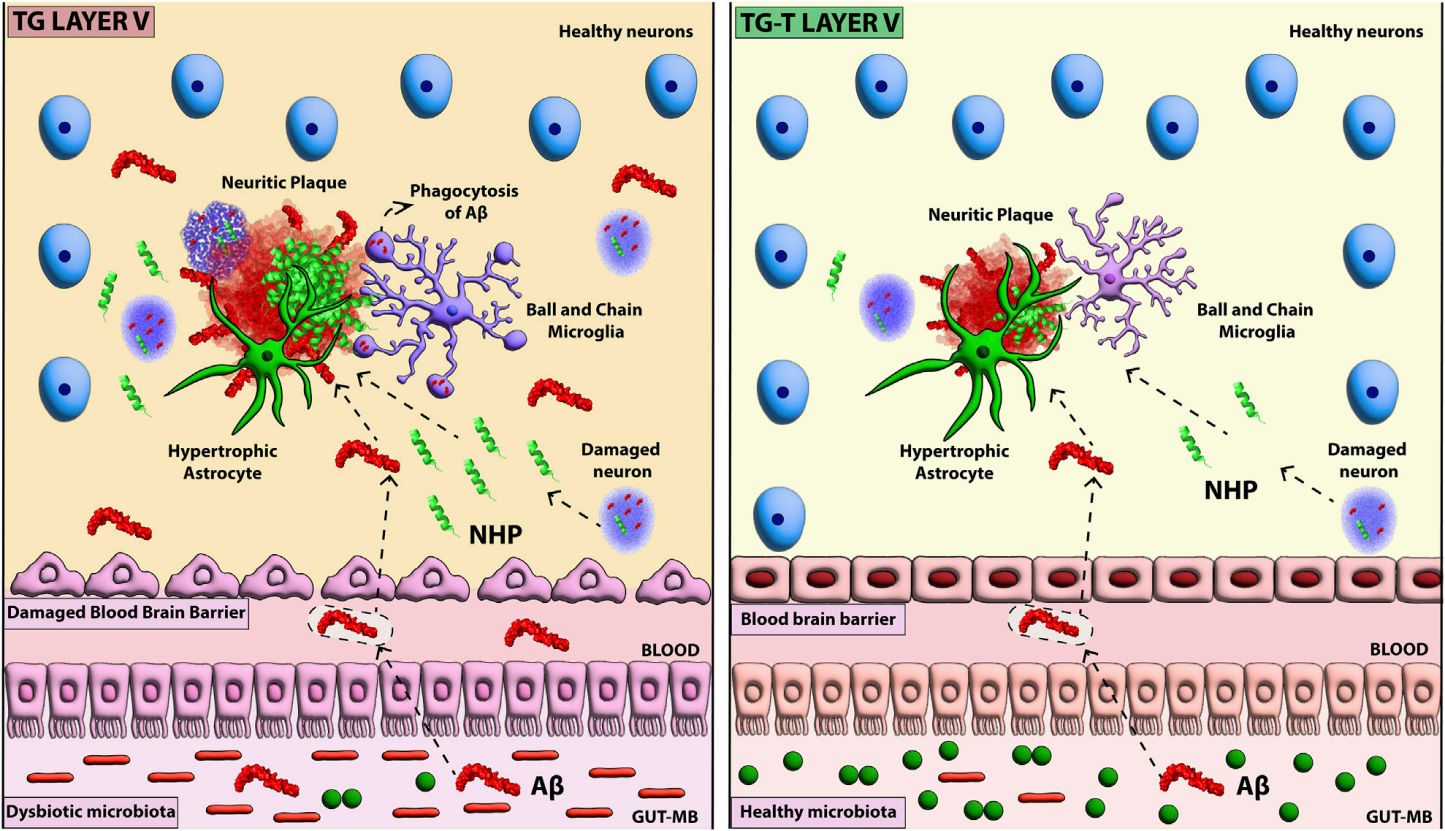
Schematic representation of the effect of pre- and probiotic administration of AD hallmarks in cortical Layer 5.

In this research, we carried out further studies on the model we recently developed of APP/PS1 mice fed with a 6-month pre- and probiotics enriched diet (Lana et al., 2024; Traini et al., 2024). APP/PS1 is a transgenic mouse strain characterized by robust and highly diffuse Aβ plaque deposition starting at 3 months of age (Radde et al., 2006). We investigated the effects of the pre- and probiotics enriched diet on the histopathological hallmarks of AD in the frontoparietal cortex of APP/PS1 mice focusing on neuronal degeneration and glia activation. In 8-month-old transgenic mice, we found intense Aβ plaque load and accumulation of neurofilament heavy polypeptide (NHP) in degenerating plaque neurites (hallmarks of AD), but also neuronal degeneration, shrinkage of the cortex, and microglia and astrocytes activation. All these effects were mainly evident in cortical Layer 5. Symbiotic treatment for 6 months with pre- and probiotics decreased Aβ deposition and neuritic plaques in the frontoparietal cortex. In addition, the treatment prevented the degeneration of neurons, the cortical shrinkage, increased GFAP expression, and modified microglia phenomic, decreasing significantly microglia activation. The abovementioned effects of the treatment were mostly evident in cortical Layer 5. All these data confirm that a prolonged dietary regimen enriched with pre- and probiotics counteracts many of the histopathological hallmarks of AD, and pose the bases for simple, affordable treatment that may help prevent AD. A schematic representation of our results is presented in Figure 8.

One of the histopathological hallmarks of AD is the accumulation of Aβ fibrils and plaques in brain parenchyma (Koyama et al., 2012; Hayden and Teplow, 2013). However, the exact origin of excessive Aβ load in the brain of animal models of AD and of AD patients has not been completely understood. Evidence suggests that both the vagal pathway (Willyard, 2021), and the increased delivery of Aβ to the brain caused by high Aβ levels in circulating blood might be responsible for increased Aβ levels in brain parenchyma during aging (Rocchi et al., 2009). Indeed, it has been demonstrated that cerebral Aβ load correlates with plasma concentration of Aβ (Nakamura et al., 2018; Jin et al., 2023). Gut microbiota and enterocytes are the major producers of Aβ outside the brain (Pallebage-Gamarallage et al., 2012; Nakamura et al., 2018; Galloway et al., 2019). The quantitative and qualitative modification of gut microbiota, mainly characterized by the increase of Gram-negative and the decrease of Gram-positive bacteria, causes aberrant accumulation of APP in the gut (Brandscheid et al., 2017; Kowalski and Mulak, 2019). In particular, *B. subtilis* (Gram+) and *E. coli* (Gram-) are the main producers of Aβ (de Kivit et al., 2014; Pistollato et al., 2016; Friedland and Chapman, 2017) which, given the dysfunctionality of the intestinal barrier demonstrated in APP/PS1 mice (Ou et al., 2020), can pass easily from the gut to the circulation. Dysbiosis, which develops during aging (for ref. see Gupta et al., 2024) and in AD patients even at the early stages of the disease (Cattaneo et al., 2017; Vogt et al., 2017; Kesika et al., 2021), may cause increased blood Aβ concentration. The altered microbiota of APP/PS1 transgenic mice overproduces Aβ (Harach et al., 2017), and causes a high Aβ load in the brain (Radde et al., 2006). Indeed, in gut autopsies of Alzheimer patients, it has been detected aberrant deposition of Aβ (Honarpisheh et al., 2020). Thus, it seems that with age or at early stages of AD, more than the vagal pathway (Willyard, 2021), the circulation route plays a major role in increasing the Aβ load in the brain (Jin et al., 2023). We had previously demonstrated that *Akkermansiaceae* were significantly reduced in gut faeces of APP/PS1 mice (Traini et al., 2024). The decrease of *Akkermansia* has been associated with intestinal barrier dysfunction, obesity, type 2 diabetes, and other metabolic syndromes which represent risk factors for AD (Cani et al., 2022). It has previously been shown that *Akkermansia* administration to APP/PS1 mice is associated with significant reduction of Aβ load in the cortex and amelioration of cognitive deficits (Ou et al., 2020). Our results suggest that a precocious 6-months treatment with pre- and probiotics, a symbiotic mixture composed of a multi-extract of fibers, plant complexes and Lactobacilli, can counteract the reduction in *Akkermansia* observed in APP/PS1 mice (Traini et al., 2024). Additional investigations are needed to understand the mechanisms underlying the modulation of the microbiota in APP/PS1 mice by the symbiotic administration of pre- and probiotic. In this respect, *Akkermansia* has been demonstrated to reduce levels of Aβ40-42, to normalize BDNF and improve cognitive functions in mice (Ou et al., 2020; Cheng et al., 2021).

In the present work we also studied the pattern of accumulation of neurofilament heavy polypeptide (NHP) in neurofibrillary tangles and degenerating neuritic plaques, hallmarks of AD. Indeed, AD is characterized not only by the extracellular deposition of Aβ, but also by the accumulation of Tau protein within neurons (neurofibrillary tangles), dendrites (neuropil threads), and neuritic plaques in brain parenchyma, which induce defects of synaptic connections. It has been demonstrated that neuritic plaques colocalize with Aβ deposits (Lewczuk et al., 2018; Moloney et al., 2021). We found that in the cortex of APP/PS1 mice, high levels of NHP were localized in neuritic plaques as well as in the cytoplasm of many neurons. According to Moloney and colleagues (Moloney et al., 2021) we found pretangles in neurons, with a perinuclear accumulation of NHP and a granular staining cytoplasmic pattern. We also found neuropil threads outside the soma, and neuritic plaques where NHP was associated with Aβ. NHP in neuron cytoplasm form neurofibrillary tangles, a possible cause of neuronal degeneration (Josephs et al., 2013). It has been demonstrated that Aβ can accumulate intraneuronally, with toxic effects on synapses and dendritic spines, both in AD brains and in transgenic mice models of the disease (Lacosta et al., 2017). Neuritic plaques are principally composed of neuron fragments, bulbous and thread-like dystrophic neurites surrounding a core of Aβ deposits (Probst et al., 1983; Moloney et al., 2021), and recent reports show that intracellular accumulation of Aβ, toxic for neurons, can cause the accumulation of NHP in neurofibrillary tangles and degenerating neuritic plaques, where it is deposited around Aβ (Lacosta et al., 2017). We found neurons in three different stages of degeneration around plaques: neurons with aggregates of Aβ in the cytoplasm, mainly close to the plasma membrane, neurons in close contact with the neuritic plaque with aggregates of Aβ and NHP, and neuronal fragments containing aggregates of Aβ and NHP and inglobated in the neuritic plaque. We speculate that neurons near plaques go through three different stages of degeneration. First, Aβ levels increase intraneuronally forming aggregates; second, Aβ-load triggers NHP accumulation; third, both Aβ and NHP cause neuronal death and their spillover to the parenchyma contribute to buildup of neuritic plaques. The symbiotic treatment with pre- and probiotics significantly attenuated the accumulation of Aβ and NHP in the cortex, mainly in Layer 5, and consequently did not cause the accumulation of intraneuronal NHP, decreased neuronal death and the buildup of further neuritic plaques.

In APP/PS1 mice, amyloid plaques develop in the cortex already at 2 months of age (Radde et al., 2006), and neurons of cortical Layer 5 are the earliest affected, causing age-dependent impairment of synaptic plasticity, synapse loss and cognitive decline (Crouzin et al., 2013; Seo et al., 2014; Colié et al., 2017; Li K. et al., 2019). In our experiments, at 8 months, diffuse and robust Aβ plaque deposition caused shrinkage of the whole cortical thickness, while the density of the neurons in the entire cortex was unaffected. Cortical shrinkage was mainly caused by the degeneration of neurons in Layer 5, in agreement with results previously obtained in 12-month-old 5xFAD mice (transgenic mice with 5 mutations:_ APP K670_M671delinsNL (Swedish) , APP I716V (Florida) , APP V717I (London) , PSEN1 M146L (A>C) , PSEN1 L286V ), which have significant neuronal loss in Layer 5 of the frontal cortex, while the overall neuron number in the cortex is unchanged (Jawhar et al., 2012; Chen et al., 2022; Chen et al., 2023). It appears that neurons in Layer 5 of transgenic mice are the preferential target of Aβ plaques-driven neurotoxic mechanisms. Chen and colleagues (Chen et al., 2022) demonstrated in 5xFAD mice that the neurons of Layer 5 have decreased excitatory and increased inhibitory cortical connectivity, which the authors suggest may make these neurons more vulnerable to the overexpression of APP and PS1 in this mouse strain (Chen et al., 2022). Interestingly, the 6-months long treatment with pre- and probiotics prevented not only neuritic plaque deposition, but also neuronal degeneration and cortical shrinkage. In TG-T mice, both these parameters did not differ from those of WT mice. We have previously demonstrated that APP/PS1 mice at 8 months of age have a significant increase in plasmatic Aβ1-42 concentration (Traini et al., 2024), and treatment with pre- and probiotics prevents not only gut dysbiosis, but also the increase of Aβ blood levels (Traini et al., 2024), with consequent decrease of Aβ plaque load in the hippocampus (Lana et al., 2024) and in the cortex, as here demonstrated.

In addition, upon the hypothesis that dysfunctional microbiota, which is known to influence the activation of glia (Erny et al., 2015; Rothhammer et al., 2016), we analyzed astrocytes and microglia in the cortex of the 4 experimental groups. Astrocytes density and expression of GFAP increased in the cortex of TG mice, mainly in Layer 5. The treatment with pre- and probiotics did not change the density of astrocytes, but further increased these GFAP expression, an indication that astrocytes were more activated in TG-T than in TG mice, an effect that was more significant in Layer 5.The microbiota produces aryl hydrocarbon (AH) ligands, that bind to AH receptors (AHR) located on astrocytes (Zelante et al., 2013; Rothhammer et al., 2016), from dietary tryptophan. AHs modulate the activity of astrocytes towards an anti-inflammatory phenotype (Wikoff et al., 2009; Zelante et al., 2013), promoting outgrowth and survival of neurons, synaptogenesis, as well as phagocytosis (Liddelow and Barres, 2017), and contributing to neuroprotection. Reactive astrocytes infiltrate and envelope Aβ deposits with their processes (Perez-Nievas and Serrano-Pozo, 2018), exhibit high phagocytic activity and activate autophagy (Yue and Hoi, 2023). The activation of astrocytes is no longer exclusively considered a negative phenomenon, but rather a part of a new concept recognizing the complex and diverse roles that astrocytes play in neuropathological disorders (Verkhratsky et al., 2013; Burda and Sofroniew, 2014). The gut microbiota can modulate and suppress the inflammatory state of astrocytes, with important consequences for neuroinflammation (Rothhammer et al., 2018). As in the CA3 hippocampus of TG-T mice (Lana et al., 2024), astrocytes acquired a protective phenotype also in Layer 5 of the cortex, possibly cooperating with reactive microglia in the scavenging of Aβ plaques, as also found in TgCRND8 (transgenic mice with 2 mutations: APP K670_M671delinsNL (Swedish), and APP V717F (Indiana), a different mouse model of AD (Ugolini et al., 2018). Decreased production of SCFAs from microbiota has been found in neurodegenerative diseases (Unger et al., 2016), but prebiotics can increase SCFAs production (Wong et al., 2006). The latter are also in close interaction with gut cells via AHR activation, alleviating neuroinflammation in 5xFAD transgenic mice (Salminen, 2023). An in-depth characterization of the mechanisms that control astrocytes and the role of prebiotics and probiotics on their phenotypic modification is still lacking.

In the cortex of TG mice, the density of microglia increased mostly in Layer 5, microglia cells were highly activated, and expressed a CD68- and CX3CR1-positive, ball&chain phagocytic phenotype. Activated ball&chain microglia were mainly distributed around plaques, and phagocytosed Aβ. Erny and coworkers (Erny et al., 2015) demonstrated that the microbiota is crucial for the maintenance of microglia, ready to respond rapidly to damaging stimuli such as Aβ accumulation. Signals from the microbiota delineate microglia phenomic and shape the brain innate immune system, conditioning the maturation and function of microglia (Erny et al., 2015), which have a fundamental role in synaptic circuits and cognitive functions (Kettenmann et al., 2013). Dysbiosis can cause modifications and dysfunctionality of microglia, contributing downstream to pathological alterations and dysfunctions of neurons that may lead to neurodegeneration (Palop et al., 2007; Krasemann et al., 2017). Sustained activation of microglia can increase Aβ deposition and phagocytosis of healthy neurons (Neher et al., 2011; Neher et al., 2012; Sierra et al., 2013; Brown and Neher, 2014; Vilalta and Brown, 2018), thus intensifying neurodegeneration (Deleidi et al., 2015). Interestingly, here we demonstrated that the treatment with pre- and probiotics prevented the development of the CD68- and CX3CR1-positive, ball&chain phagocytic phenotype of microglia. Indeed, blocking phagocytosis may be beneficial to spare neuronal disruption in many neurodegenerative conditions (Neher et al., 2011). It had been previously demonstrated in aged Wistar rats that a mixture of probiotics supplementation increases the expression of genes related to neuronal plasticity such as brain derived neurotrophic factor (BDNF), improves age-dependent deficits in long term potentiation (LTP), and decreases the expression of markers of microglia activation such as CD68 and CD11b (Integrin αM) (Distrutti et al., 2014). Interestingly, immune cells that express receptors for SCFAs derived from the microbiota can pass through the BBB and enter the brain where they can have a protective function (Wang et al., 2018). Microglia reactivity is a highly regulated, multistaged and reversible process that generates multiple phenotypes with protective or damaging capacity (Hanisch and Kettenmann, 2007; Ransohoff and Perry, 2009; Glass et al., 2010; Kettenmann et al., 2013; Lana et al., 2023). Microglia maintain their protective role during normal aging (Faulkner et al., 2004; Myer et al., 2006; Block et al., 2007; Hanisch and Kettenmann, 2007; Li et al., 2008) but this ability is considerably decreased in proinflammatory contexts in which microglia play a critical role in maintaining inflammatory responses (Glass et al., 2010). In low-grade inflammatory conditions, microglia patrol brain parenchyma to reduce the spreading of inflammation, preventing further damage to neighboring neurons (Cerbai et al., 2012; Lana et al., 2016). Indeed, in an animal model of AD the decrease of phagocytic activity and clearance capacity of microglia inversely correlate with Aβ plaque deposition (Krabbe et al., 2013). Microglia located in different brain areas respond in a different way to the same insult such as plaque deposition, indicating that the changes of their phenotypic states are independent from the insult, but respond to internal, spatial and temporal dependent cues (Zhang and Barres, 2010; Cerbai et al., 2012; Khakh and Sofroniew, 2015; Ben Haim and Rowitch, 2016; Lana et al., 2016; Liddelow and Barres, 2017; Khakh and Deneen, 2019; Pestana et al., 2020). Many papers in the literature are demonstrating that, in response to a damaging stimulus, microglia can acquire different spatiotemporal functional phenotypes. The phagocytic activity and clearance capacity of microglia inversely correlate with Aβ plaque deposition and aging (Blume et al., 2018). Microglia responses in AD are influenced by apolipoprotein E (APOE) and Triggering receptor expressed on myeloid cells 2 (TREM2) (Krasemann et al., 2017). TREM2 regulates microglia energetic and biosynthetic metabolism (Ulland et al., 2017), maintaining their high activity to dispose of excess Aβ. However, the intense TREM2-dependent microglia activation during time can cause harmful chronic inflammatory responses (Krasemann et al., 2017). Understanding the differences of microglia phenomic states, their temporal and spatial distribution, and reactivity may help explain the differential susceptibility of different brain areas to the same inflammatory insult such as plaque deposition and to the attenuating effects of pre- and probiotic administration (Masgrau et al., 2017; De Felice et al., 2022).

In a previous paper (Traini et al., 2024) we demonstrated that the treatment with pre- and probiotics was able to attenuate the memory deficits in cortically-mediated consolidation of inhibitory avoidance (Pereira et al., 2001), which provide a clear link between the phenomic changes in the cerebral cortex and the cognitive outcomes in this animal model.

Although it is generally accepted that the cortex functions as a complex and interconnected network of cells, it is unknown whether specific areas are more vulnerable than others, showing selective (affecting a single region), preferential (greater in one region than in another), or general (affecting the entire cortex) degeneration (Salat et al., 2001). Layer 5 (L5) serves as the main output layer of cortical structures, where long-range projecting pyramidal neurons broadcast the columnar output to other cortical and extracortical regions of the brain. As previously reported (Crouzin et al., 2013; Seo et al., 2014; Colié et al., 2017; Li N. et al., 2019), here we found that not only neurons, but also astrocytes and microglia in cortical Layer 5 are the most affected cells in TG mice, evidencing a selective process that involves plaques deposition, glia reactivity, neurodegeneration and cortical shrinkage. It is interesting to note that in response to the symbiotic treatment, microglia and astrocytes changed their morphological features in a Layer-specific way, indicating once more that these cells are composed of heterogeneous populations, which differ in their phenomic responses to damaging and/or protective stimuli. Indeed, most of the effects found in both TG and TG-T mice were distributed mainly in Layer 5 of the cortex.

It must be pointed out that the symbiotic treatment with pre- and probiotics attenuated memory deficits in this transgenic mouse model (Traini et al., 2024). Recent data demonstrate that acting on dysbiotic microbiota, an important source of Aβ outside the brain, could be the key to prevent or attenuate Alzheimer’s disease pathophysiological modifications (Selkoe and Hardy, 2016; Samaey et al., 2019; Giovannini et al., 2021). These data further demonstrate that a healthy microbiota can positively influence brain health, posing the bases for a simple, affordable treatment that may help prevent AD.

## Data Availability

The raw data supporting the conclusions of this article will be made available by the authors, without undue reservation.
